# Inhaled Angiopoietin‐Like 4 Antisense Oligonucleotide Therapy for Lung Injury and Fibrosis

**DOI:** 10.1002/advs.202501909

**Published:** 2026-03-23

**Authors:** Haiyang Fan, Yuanyang Tan, Junhang Zhang, Xiaoya Liu, Jing Qu, Damien Chua, Hong Sheng Cheng, Joseph Han Sol Kim, Yu Xuan Liu, Changfei Qin, Yingzi Liu, Dezhi Li, Jikang Qiu, Mengshi Chi, Mingmin Bi, Qiwei Zhang, Yun Li, Masoumeh Motamedi Joibari, Stefan K Nilsson, Nguan Soon Tan, Yunping Fan, Liang Li

**Affiliations:** ^1^ Department of Otolaryngology The Seventh Affiliated Hospital, Sun Yat‐Sen University Shenzhen P. R. China; ^2^ Department of Thoracic Surgery The Seventh Affiliated Hospital, Sun Yat‐Sen University Shenzhen P. R. China; ^3^ Joint Laboratory of Guangdong‐Hong Kong Universities for Vascular Homeostasis and Diseases, Department of Pharmacology School of Medicine Southern University of Science and Technology Shenzhen P. R. China; ^4^ Department of Pharmacy Shenzhen Children's Hospital Shenzhen Guangdong Province 518026 P. R. China; ^5^ Shenzhen Center for Disease Control and Prevention Shenzhen 518055 P. R. China; ^6^ Lee Kong Chian School of Medicine Nanyang Technological University Singapore 11 Mandalay Road Singapore 308232 Singapore; ^7^ Department of Pathology The Seventh Affiliated Hospital Sun Yat‐Sen University Shenzhen P. R. China; ^8^ Intervention and Cell Therapy Center Peking University Shenzhen Hospital Shenzhen P. R. China; ^9^ Department of Minimally Invasive Intervention Peking University Shenzhen Hospital Shenzhen Guangdong 518000 P. R. China; ^10^ Lipigon Pharmaceuticals AB Umeå Sweden; ^11^ School of Biological Sciences Nanyang Technological University Singapore 60 Nanyang Drive Singapore 637551 Singapore

**Keywords:** antisense therapy, angiopoietin‐like 4, host‐directed therapy, lung inflammation, lung fibrosis

## Abstract

Pulmonary infections and fibrosis remain difficult to treat because current interventions target isolated pathways rather than the coupled axes of inflammation, barrier integrity, and tissue remodeling. Here, it is shown that inhalationally delivered, lung‐targeted antisense oligonucleotides against angiopoietin‐like 4 (*Angptl4*‐ASO) attenuate both infectious and fibrotic lung disease. In murine models of bacterial and viral pneumonia, *Angptl4*‐ASO reduces inflammatory cell infiltration, preserves alveolar architecture, and improves host defence. In bleomycin‐induced fibrosis, treatment lowered Ashcroft scores, collagen deposition, and α‐smooth muscle actin (SMA) expression, indicating broad efficacy across acute and chronic injury. Comparative transcriptomics reveal model‐specific responses, immune and oxidative‐stress programs in pneumonia versus extracellular matrix (ECM)‐remodeling pathways in fibrosis, yet nearly half of all changes converge on a shared ANGPTL4‐regulated network linking hypoxic, inflammatory, apoptotic, and stress response programs. This conserved signature suggests that ANGPTL4 functions as a central regulator of injury resolution regardless of the initiating insult. Mechanistically, *Angptl4*‐ASO reinforced epithelial barrier integrity through coordinated regulation of tight junction and glycoprotein pathways. Longitudinal tracking of a Sulfo‐Cyanine 5 (Cy5)‐conjugated *Angptl4*‐ASO confirmed a lung‐retentive biodistribution, with sustained intrapulmonary localization and minimal systemic dissemination over a 144‐hour window. Collectively, these findings position inhaled ANGPTL4‐ASO as a host‐directed, multi‐axis therapeutic strategy that addresses shared and context‐specific drivers of diverse pulmonary pathologies.

## Graphical abstract

Inhaled antisense oligonucleotides targeting ANGPTL4 attenuate inflammation, preserve barrier integrity, and reduce fibrosis in both infectious and sterile lung injury models. Integrated transcriptomic analysis reveals a conserved ANGPTL4‐regulated gene network coordinating hypoxic, inflammatory, apoptotic, and stress‐response pathways. This work establishes a lung‐retentive, host‐directed therapeutic strategy that targets shared and context‐specific drivers of pulmonary disease — spanning viral, bacterial, and idiopathic respiratory conditions as well as fibrosis.

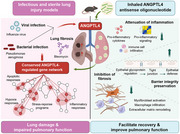

## 1 Introduction

Acute lung injury (ALI) and its severe aftermath, pulmonary fibrosis, are major clinical challenges in intensive care. Frequently arising from respiratory infections, these conditions reflect a dynamic interplay between pathogen‐induced inflammation and tissue remodeling.^[^ ^1^, ^2^ ^]^ The COVID‐19 pandemic highlighted the lasting impact of such injuries, with ≈21% of recovered patients showing persistent radiographic signs of fibrosis.^[^ ^3^, ^4^ ^]^ Opportunistic pathogens like *Pseudomonas aeruginosa*, common in hospital‐acquired infections, further intensify ALI and fibrosis, especially in patients with pre‐existing airway damage from mechanical ventilation, trauma, or prior viral infections. In such vulnerable patients, *P. aeruginosa* infections often lead to acute pneumonia and sepsis, escalating inflammatory injury.^[^ ^5^, ^6^ ^]^ Current therapies primarily focus on managing infections and suppressing inflammation through antibiotics and anti‐inflammatory agents.^[^ ^7^, ^8^ ^]^ Yet, these approaches often fail to address the multifaceted nature of ALI and its progression to fibrosis, amid rising antibmicrobial resistance.

Recent work highlights angiopoietin‐like 4 (ANGPTL4) as a biomarker and therapeutic candidate in ALI and fibrosis. Elevated levels of ANGPTL4 correlate with severe outcomes in pneumonia, acute respiratory distress syndrome (ARDS), and even COVID‐19, suggesting its pivotal role in disease progression.^[^ ^9^, ^10^, ^11^, ^12^ ^]^ Structurally, ANGPTL4 comprises a 15 kDa N‐terminal coiled‐coil domain and a 35 kDa C‐terminal fibrinogen‐like domain (cANGPTL4) linked by a proprotein‐convertase recognition segment that enables context‐dependent post‐translational processing.^[^ ^13^ ^]^ This unique structure underpins diverse roles in lipid metabolism, angiogenesis, and inflammation.^[^ ^9^, ^14^ ^]^ Functionally, ANGPTL4 can mitigate saturated‐fat–driven inflammatory stress by limiting fatty‐acid uptake and modulating mediators such as IL‐6, underscoring therapeutic relevance in inflamed lung tissue.^[^ ^15^ ^]^

Clinical data have revealed that ANGPTL4 levels can increase more than tenfold in severe pneumonia and ARDS compared to healthy individuals.^[^ ^12^, ^16^, ^17^ ^]^ High‐throughput RNA sequencing from influenza pandemics and multi‐organ proteomic analyses in COVID‐19 cases have demonstrated significant upregulation of ANGPTL4,^[^ ^18^, ^19^ ^]^ correlating with worse clinical outcomes, including higher incidents of venous thromboembolism and increased mortality rates.^[^ ^16^ ^]^ Experimental models, spanning influenza and lipopolysaccharide‐induced lung injury, converge on increased ANGPTL4 expression, aligning with increased severity of lung damage and progression to chronic disease states.^[^ ^10^, ^11^, ^20^ ^]^

Together, these observations position ANGPTL4 as a central coordinator of lung injury biology across infectious and fibrotic contexts. However, two critical gaps remain: (i) a lack of host‐directed, lung‐targeted interventions that simultaneously modulate inflammation, preserve epithelial/vascular barriers, and interrupt fibrotic remodeling; and (ii) the challenge of precisely modulating ANGPTL4 in disease tissue without disrupting its context‐dependent beneficial functions. RNA‐based therapeutics offer a path forward. Building on advances exemplified by SARS‐CoV‐2 mRNA vaccines,^[^ ^21^, ^22^ ^]^ antisense oligonucleotides (ASOs) provide high specificity, access to traditionally “undruggable” proteins, and adaptability in design. These characteristics position ASOs as promising candidates for managing inflammatory conditions by regulating cytokine expression and influencing protein translation and stability.^[^ ^23^, ^24^ ^]^

To address this gap, we investigate ANGPTL4‐targeting ASOs in models of infection‐triggered ALI and bleomycin‐induced fibrosis. We hypothesized that by targeting ANGPTL4, we can simultaneously decrease pathogen‐induced inflammation and halt the progression to fibrosis. This approach could potentially revolutionize the treatment strategy for ALI and its complications, offering a more comprehensive solution than current therapies.

## 2 Results

### 2.1 *ANGPTL4*‐ASO is a Potent Modulator of Hypoxia‐Induced ANGPTL4 Expression

To assess the efficacy of ANGPTL4‐targeting antisense oligonucleotides (ANGPTL4‐ASO), we conducted in vitro tests using synthetic ASOs specific to both human and mouse *ANGPTL4*. These tests were performed on HaCat (human keratinocytes) and AML12 (mouse hepatocytes) cell lines. The findings demonstrate that *ANGPTL4*‐ASOs significantly suppress the elevated expression of *ANGPTL4* under hypoxic conditions, a key pro‐survival response in various pathological settings.^[^ ^25^, ^26^ ^]^ Hypoxia, a hallmark of pulmonary fibrosis (PF), triggers a dramatic increase in *ANGPTL4* expression as part of the cellular stress response.^[^ ^27^ ^]^

Hypoxia represents a key microenvironmental signal during acute and fibrotic lung injury, arising from impaired ventilation, vascular leakage, and inflammatory cell infiltration. These conditions activate the HIF‐1α pathway, a central transcriptional regulator of cellular adaptation to low oxygen. Stabilized HIF‐1α directly binds the hypoxia response element (HRE1; ‐206 to ‐202 bp) within the *ANGPTL4* promoter, promoting its transcription under hypoxic stress^[^ ^28^ ^]^. This mechanism links tissue hypoxia to *ANGPTL4* induction observed in injured lungs. Thus, the HIF‐1α‐*ANGPTL4* axis provides a mechanistic basis for the elevated *ANGPTL4* expression seen in our lung injury models.

**Figure 1:**
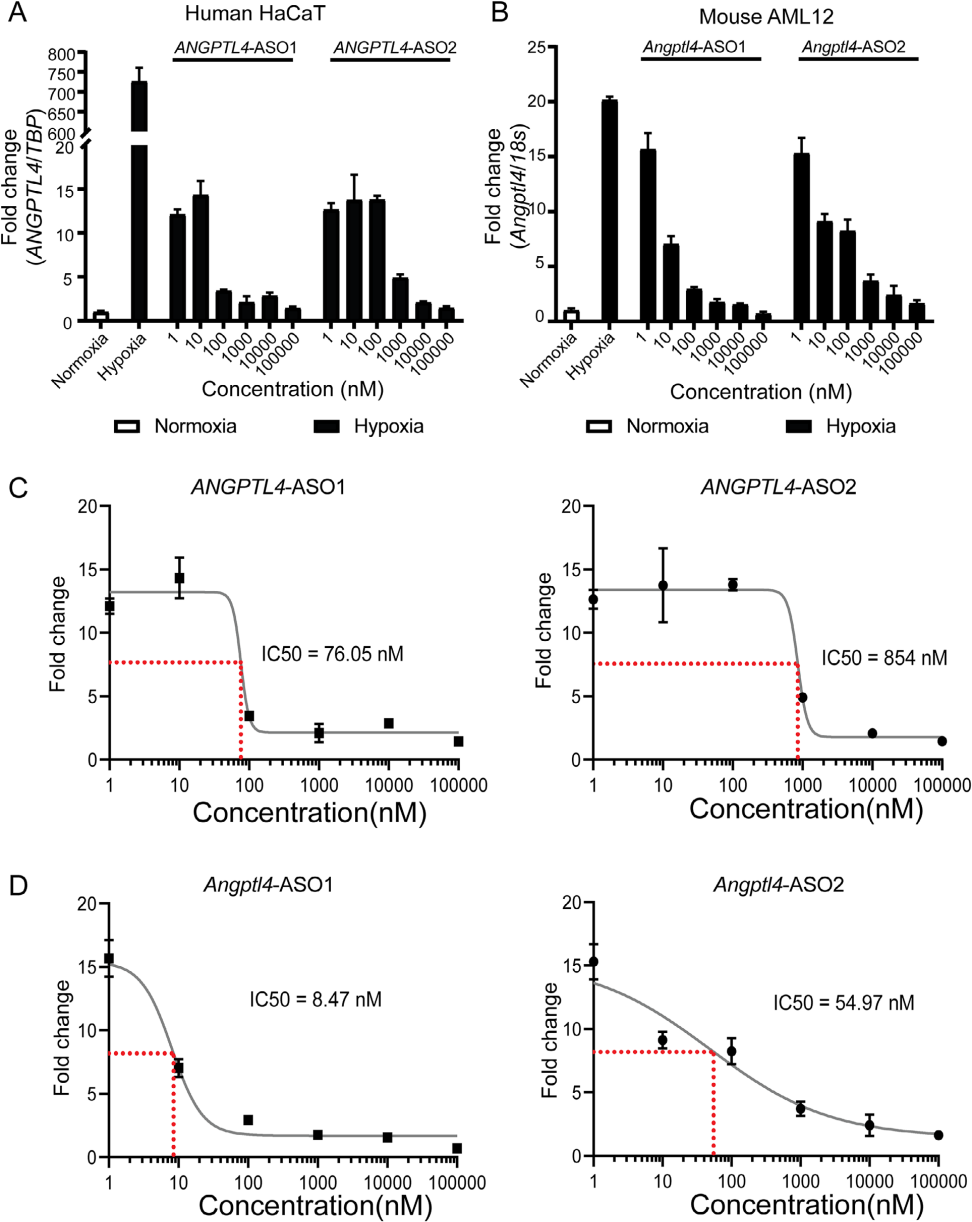
In vitro validation of *ANGPTL4*‐ASO knockdown efficacy in human and mouse cell lines. A,B) Dose‐dependent suppression of *ANGPTL4* expression in human HaCaT keratinocytes A) and mouse AML12 hepatocytes B) under normoxic and hypoxic conditions. Cells were treated with increasing concentrations (1–100 000 nM) of two *ANGPTL4*‐ASOs. Gene expression was normalized to *TBP* (human) or 18S (mouse) and presented as fold change relative to normoxia. C,D) Dose‐response curves and IC50 determinations for human *ANGPTL4*‐ASOs in HaCaT cells C) and mouse *Angptl4*‐ASOs in AML12 cells D) under hypoxic conditions. IC50 values: human *ANGPTL4*‐ASO1 (76.05 nM), *ANGPTL4*‐ASO2 (854 nM); mouse *Angptl4*‐ASO1 (8.47 nM), *Angptl4*‐ASO2 (54.97 nM). Error bars represent Standard Deviation (SD). IC50 values were calculated using non‐linear regression analysis.

In this context, *ANGPTL4*‐ASOs displayed a potent, dose‐dependent inhibition of hypoxia‐induced *ANGPTL4* upregulation (**Figure** **1**A,B). Notably, the ASOs achieved sub‐micromolar half‐maximal inhibitory concentration (IC50) values, indicating high efficiency at low concentrations. Human‐targeting ASOs exhibited IC50 values of 76 and 854 nM, while mouse‐targeting ASOs demonstrated even greater potency with IC50 values of 8.47  and 54.97 nM (Figure 1C,D). These results underscore the robust inhibitory potential of *ANGPTL4*‐ASOs and their applicability across species. By effectively reducing *ANGPTL4* expression under hypoxic stress, these ASOs emerge as a promising therapeutic strategy for conditions driven by hypoxia‐induced *ANGPTL4* overexpression, such as pulmonary fibrosis.

### 2.2 *Angptl4*‐ASO Reduces Lung Inflammation and Injury in Murine Lung Infections

Severe respiratory infections, such as those caused by *Pseudomonas aeruginosa* (PAO1), often lead to acute lung injury (ALI) characterized by excessive inflammation and tissue damage. Given the established role of ANGPTL4 in exacerbating inflammatory responses, we hypothesized that targeted suppression of *Angptl4* using ASO could mitigate lung inflammation and reduce tissue injury during bacterial infections.

**Figure 2:**
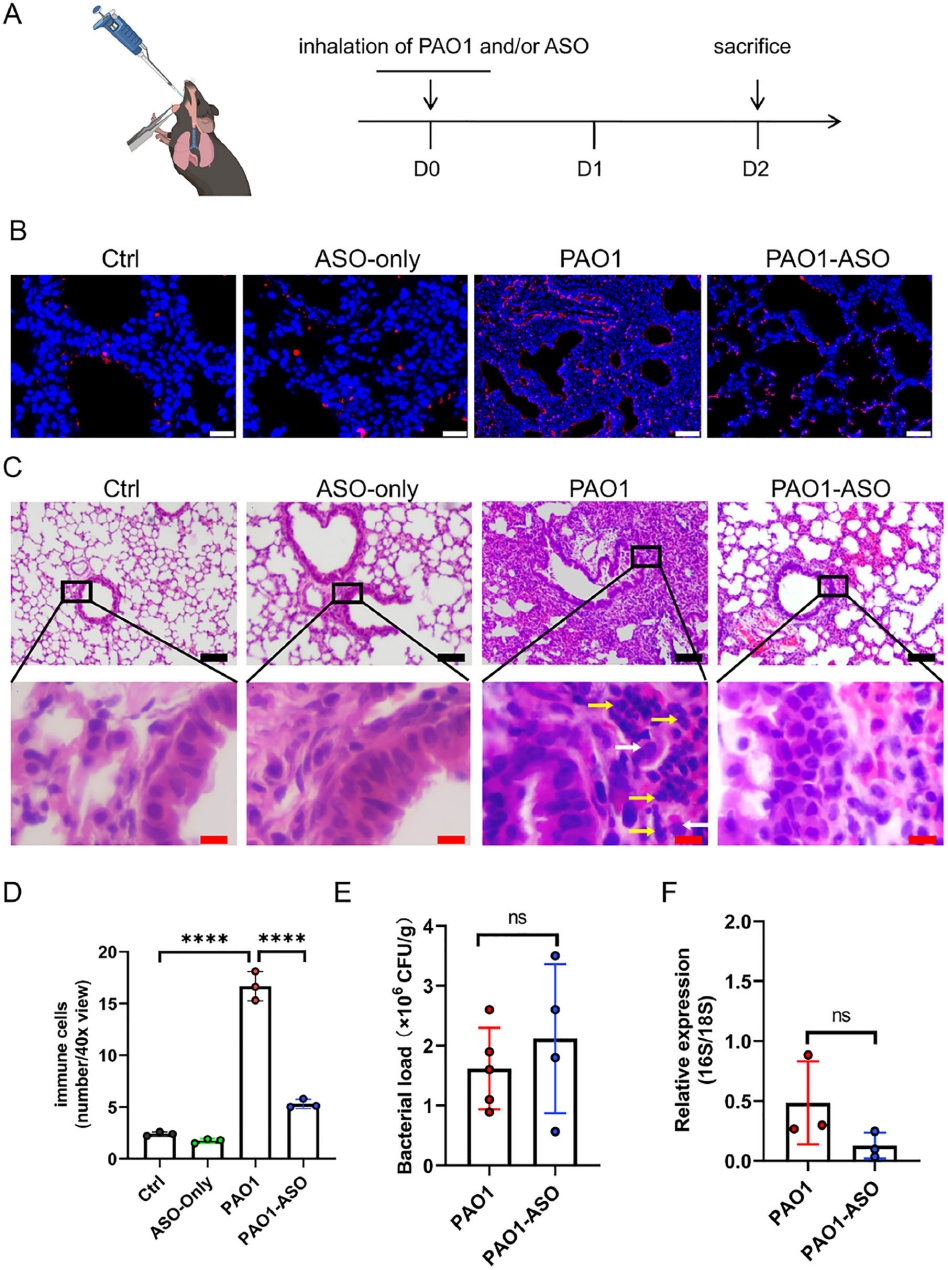
*Angptl4*‐ASO reduces lung inflammation and tissue damage in PAO1 infection. A) Experimental timeline of the *P. aeruginosa* (PAO1) lung infection mouse model. D0: PAO1 and/or *Angptl4*‐ASO inhalation; D2: sacrifice. B) Immunofluorescence staining of ANGPTL4 (red) in mouse lung sections. Nuclei were counterstained with DAPI (blue). Ctrl and ASO‐only groups show minimal ANGPTL4 expression in bronchial epithelium and walls. PAO1 group exhibits significant ANGPTL4 expression in bronchial epithelium, surrounding areas, lung interstitium, and alveoli. PAO1‐ASO group shows significant downregulation compared to PAO1 group (scale bar: 20 µm). C) Hematoxylin and eosin (H&E) staining of mouse lung sections. Control and ASO‐only treated lungs (a‐d) show no inflammatory exudation or cellular infiltration. PAO1 treated lungs (e, f) display severely damaged lung alveolar structure and extensive cellular infiltration (yellow arrows indicates neutrophils; white arrows indicates macrophages). PAO1‐ASO treated lungs (g, h) exhibit notable recovery from tissue injury, preserved alveolar structure, and reduced immune cell infiltration (Black scale bar: 100 µm; Red scale bar: 10 µm). D) Quantification of inflammatory cells in alveoli (40× field of view). PAO1 group shows significantly higher inflammatory cell counts compared to all other groups. PAO1‐ASO group exhibits reduced inflammation compared to PAO1 group but elevated compared to Ctrl. Data are shown as mean ± SD (*n* = 3 per group). Statistical comparisons were performed using one‐way ANOVA followed by Tukey’s test (****p < 0.0001 ). E) Bacterial load in lung tissue. No significant difference in CFU/g between PAO1 and PAO1‐ASO groups. Data are shown as mean ± SD (*n* = 4 or 5 per group). Statistical comparisons were performed using two‐tailed unpaired Student's *t*‐test (ns, *p* > 0.05). F) Relative expression of PAO1 16S mRNA. No significant difference between PAO1 and PAO1‐ASO groups. Data are shown as mean ± SD (*n* = 3 per group). Statistical comparisons were performed using two‐tailed unpaired Student's *t*‐test (ns, *p* > 0.05).

To test this hypothesis, we used a well‐established mouse model of PAO1 lung infection to evaluate the in vivo effects of *Angptl4*‐ASO on lung inflammation, tissue integrity, and bacterial clearance. Mice were infected with PAO1 on D0 and subsequently treated with *Angptl4*‐ASO via inhalation (**Figure** **2**A), mimicking a clinically relevant administration route.

In uninfected mice, *Angptl4*‐ASO treatment had no adverse effects, preserving lung histology (Figure 2C). Lung tissue has intact bronchial and alveolar structures and no signs of inflammation (Figure 2C). No observable pathological changes in the liver, kidney and trachea were observed (Figure S1A,B, Supporting Information). In contrast, PAO1‐infected mice showed a dramatic increase in pulmonary ANGPTL4 levels (Figure 2B), accompanied by severe lung damage, significant edema, and extensive infiltration of neutrophils and macrophages, hallmarks of ALI (Figure 2C). The Wet/Dry (W/D) ratio of lung lobes confirmed pulmonary edema in infected animals, which was significantly improved by *Angptl4*‐ASO (Figure S1C, Supporting Information). Treatment with *Angptl4*‐ASO significantly reduced infection‐induced ANGPTL4 protein expression and inflammation‐associated pulmonary damage (Figure 2B,C,D). Consistent with the immunofluorescence results, protein quantification confirmed a marked increase in ANGPTL4 expression in the lungs of PAO1‐infected mice, which was effectively reduced following *Angptl4*‐ASO treatment (Figure S1D, Supporting Information). Neutrophil and macrophage infiltration decreased 3‐fold in treated mice compared to untreated counterparts (Figure 2D). Furthermore, there was a trend toward reduced bacterial burden in the *Angptl4*‐ASO‐treated group (Figure 2E,F). Moreover, analysis of bronchoalveolar lavage fluid (BALF) from PAO1‐infected mice revealed that pro‐inflammatory cytokines, including CCL4, CCL5, CCL7, CCL20, CCL24, and IL‐6, were significantly reduced in *Angptl4*‐ASO–treated animals relative to the untreated group (Figure S2, Supporting Information).

To better reflect clinical dosing patterns, we established a multiple administration model of PAO1 infection. Mice were infected with PAO1 on Day 0 and Day 5, and received inhaled ASO on the same days. All mice were euthanized, and samples were collected on D10 for analysis (Figure S3A, Supporting Information). Histopathological analysis consistently demonstrated mitigation of lung inflammation with *Angptl4*‐ASO (Figure S3B, Supporting Information). Flow cytometric analysis of single‐cell suspensions from lung tissues showed a significant reduction in the proportion of neutrophils among leukocytes in treated versus untreated PAO1‐infected mice (Figure S3C, Supporting Information). Correspondingly, composite health scores were significantly improved in the *Angptl4*‐ASO group, indicating accelerated recovery (Figure S3D, Supporting Information).

These findings demonstrate that *Angptl4*‐ASO effectively dampens excessive inflammation, accelerates resolution of the inflammatory response, and protects lung tissue from damage, a critical determinant of disease severity and recovery. By targeting both immediate inflammation and the long‐term tissue injury associated with acute lung infection, ANGPTL4‐ASO shows significant potential as a therapeutic candidate for severe respiratory infections and their complications.

While our findings demonstrated the efficacy of *Angptl4*‐ASO in bacterial pneumonia, respiratory viral infections remain a significant clinical challenge. We hypothesized that *Angptl4*‐ASO could also reduce inflammation and lung injury in viral infections, offering a broad‐spectrum therapeutic approach for acute respiratory diseases. To test this hypothesis, we utilized a well‐established influenza PR8 virus infection model, known to cause severe lung inflammation and tissue injury.

**Figure 3:**
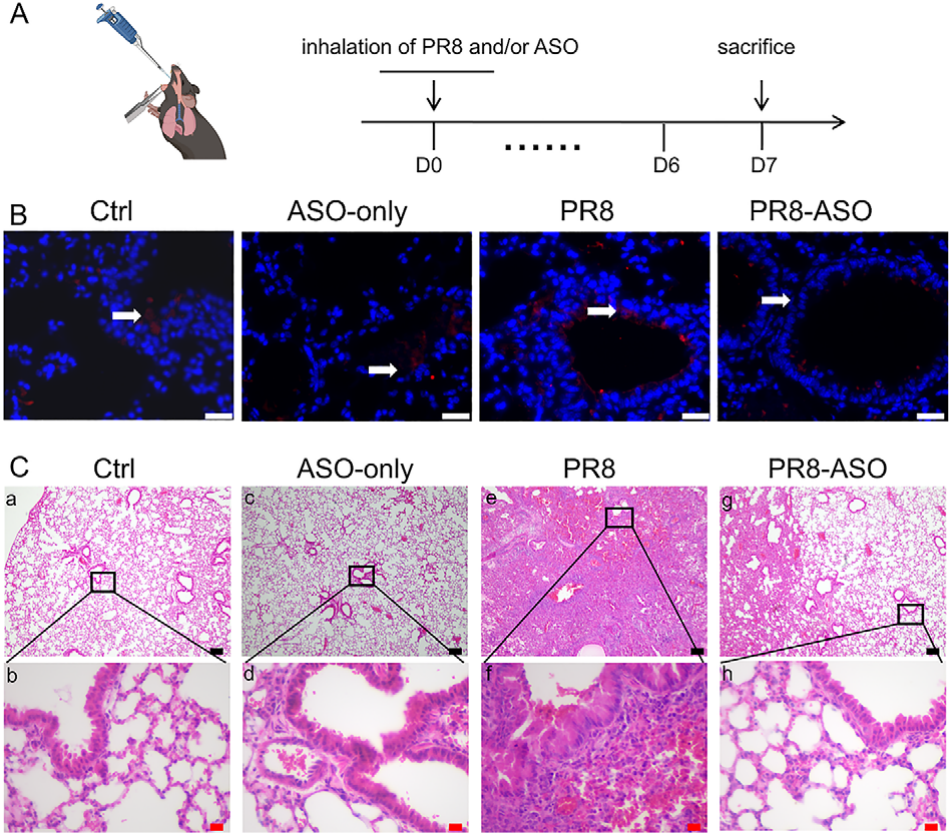
*Angptl4*‐ASO treatment reduces lung inflammation in PR8 influenza infection. A) Experimental timeline of the Influenza virus PR8 lung infection mouse model. D0: PR8 and/or *Angptl4*‐ASO inhalation; D7: sacrifice. B) Immunofluorescence staining of ANGPTL4 (red) in mouse lung sections. Nuclei were counterstained with DAPI (blue). Ctrl and ASO‐only groups show minimal ANGPTL4 expression in bronchial epithelium and walls. PR8 group exhibits significant ANGPTL4 expression in bronchial epithelium, surrounding areas, lung interstitium, and alveoli. PR8‐ASO group shows significant downregulation compared to PR8 group, with only minimal expression in bronchial epithelium and walls (White arrows indicate sites of ANGPTL4 expression; scale bar: 20 µm). C) Representative H&E staining of mouse lung sections. Ctrl and ASO‐only treated lungs (a‐d) show no inflammatory exudation or cellular infiltration. PR8 treated lungs (e, f) display severely damaged lung alveolar structure and extensive cellular infiltration. PR8‐ASO treated lungs (g, h) exhibit notable relief of inflammatory response, preserved alveolar structure, and reduced cellular infiltration (Black scale bar: 100 µm; Red scale bar: 10 µm).

Our study showed that *Angptl4*‐ASO effectively alleviated lung inflammation and tissue injury during viral respiratory infections. Mice infected with the PR8 virus received *Angptl4*‐ASO via inhalation (**Figure** **3**A). By Day 7 post‐infection, lung analysis revealed reduced pulmonary ANGPTL4 levels in ANGPTL4‐ASO treated mice, as evidenced by decreased immunofluorescence signaling (Figure 3B) and protein expression (Figure S4A, Supporting Information), confirming successful target engagement and suppression. Body weight monitoring showed a beneficial treatment effect of ASO treatment, and W/D ratio measurements showed significant improvement in pulmonary edema (Figure S4B,C, Supporting Information). Histological assessments highlighted a stark contrast between treatment groups. Ctrl and ASO‐only groups displayed minimal lung damage, reaffirming the safety profile of *Angptl4*‐ASO in uninfected tissue (Figure 3C). In contrast, PR8‐infected mice exhibited severe bronchial and alveolar injury, including extensive inflammatory cell infiltration and edema, hallmarks of acute viral pneumonia (Figure 3C).

To better reflect clinical treatment approach, a multiple‐dose regimen was established. Mice were infected with PR8 on Day 0 and Day 5, and received concurrent inhaled ASO treatment. All samples were collected on Day 10 (Figure S5A, Supporting Information). Histopathological analysis demonstrated a comparable degree of pathological remission in the single‐dose model. (Figure S5B, Supporting Information). Flow cytometric analysis of single‐cell suspensions from lung tissues further revealed that *Angptl4*‐ASO treatment markedly increased the proportion of CD19⁺ B cells, a population known to exert immunomodulatory functions during viral infection (Figure S5C, Supporting Information).

Treatment with *Angptl4*‐ASO in PR8‐infected mice significantly reduced lung damage and inflammation compared to untreated controls. The marked alleviation of bronchial and alveolar injury, coupled with decreased inflammatory cell infiltration, underscores the potent anti‐inflammatory effects of *Angptl4*‐ASO in viral infection contexts. These findings suggest that *Angptl4*‐ASO holds promise as a versatile therapeutic agent for mitigating lung injury caused by diverse respiratory pathogens, including both bacteria and viruses.

### 2.3 Transcriptomic Analysis Reveals *Angptl4*‐ASO Modulation of Pro‐Inflammatory Signaling

**Figure 4:**
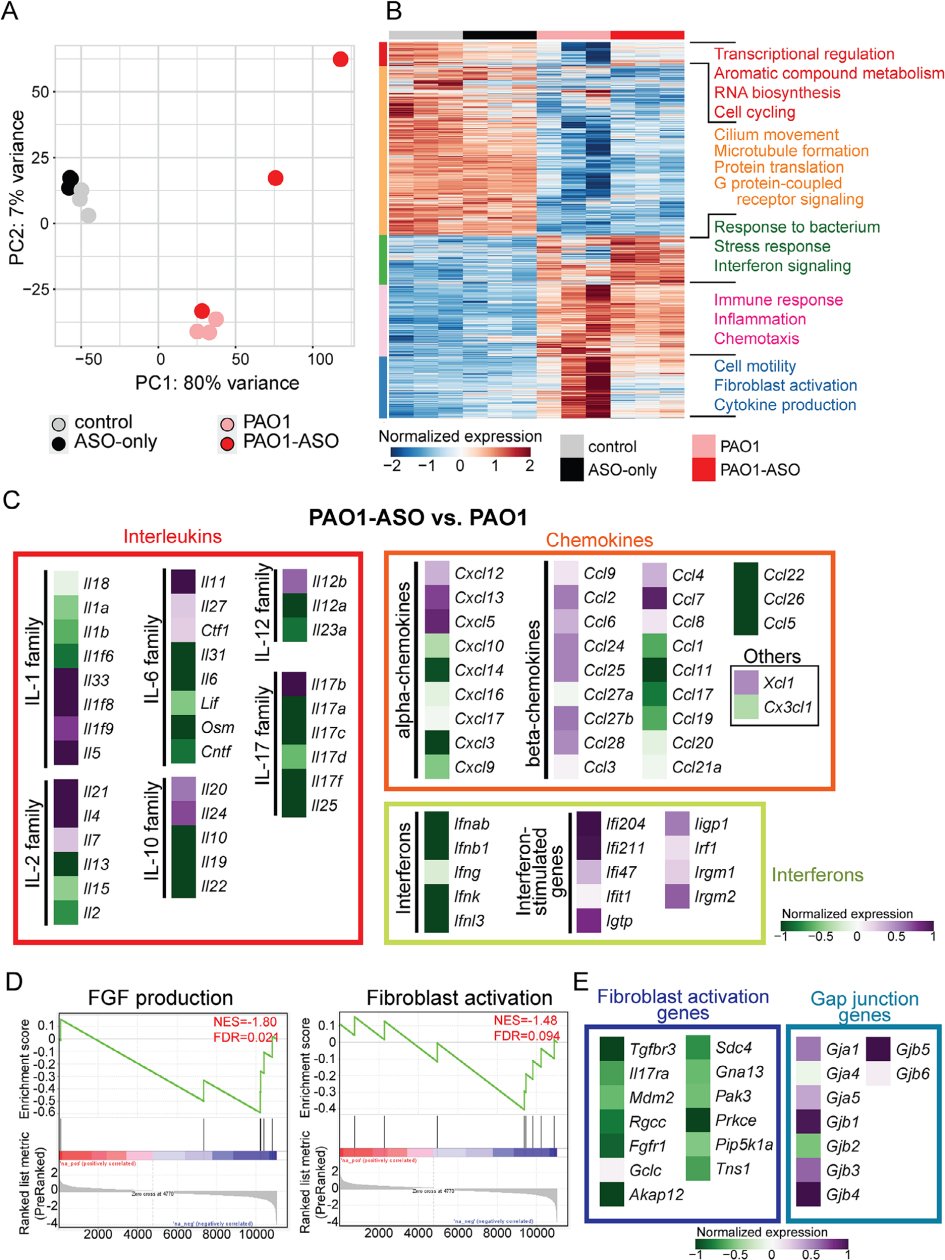
Transcriptome analysis reveals molecular activities of *Angptl4*‐ASO on day 2 post‐PAO1 infection. A) Principal component analysis (PCA) plot showing the clustering of lung transcriptomes across treatment groups (*n* = 3 per group). B) Heatmap illustrating DEGs in between treatment groups. Each row represents a gene, and each column a sample. Red indicates higher expression levels and blue indicates lower expression levels. C) Heatmap of the differential gene expression of cytokines and chemokines between *Angptl4*‐ASO treated and untreated mice infected with PAO1. The genes are grouped based on their molecular families. Purple indicates higher expression while green indicates lower expression in PAO1‐ASO relative to PAO1 mice. D) Enrichment plots of gene sets related to fibroblast growth factor (FGF) production and fibroblast activation. E) Heatmap of the differential expression of genes related to fibroblast activation and gap junction between *Angptl4*‐ASO treated and untreated mice infected with PAO1. Purple indicates higher expression while green indicates lower expression in PAO1‐ASO relative to PAO1 mice.

To elucidate the mechanism underlying *Angptl4*‐ASO's therapeutic effects, we performed lung transcriptomic profiling comparing treated and untreated groups. In uninfected mice, *Angptl4*‐ASO produced no meaningful transcriptomic changes, as reflected by the low number of differentially expressed genes (DEGs; 1 upregulated, 15 downregulated) and tight clustering pattern among the two groups in the PAO1 acute infection model analyzed on day 2 post‐infection (**Figure** **4**A; Figure S6A, Supporting Information), supporting the favorable safety profile. In contrast, PAO1 infection induced substantial remodeling of the lung transcriptome. Within the PAO1‐infected, treated and untreated mice separated clearly, consistent with the treatment effects of *Angptl4*‐ASO (Figure 4A).

Compared to untreated PAO1‐infected mice, *Angptl4*‐ASO treatment led to 29 upregulated and 102 downregulated DEGs functionally linked to activation of stress response pathways and suppression of immune signaling, cytokine production and fibrogenic activity (Figure 4B; Figure S6A, Supporting Information). Pro‐inflammatory cytokine families, including IL‐12 and IL‐17, were reduced, as were interferons (e.g., *Ifnab*, *Ifnb1*, *Ifnk, and Ifnl3*) and selected α‐chemokines (e.g., *Cxcl3* and *Cxcl14*) and β‐chemokines (e.g., *Ccl5*, *Ccl11*, *Ccl22*, and *Ccl26*) (Figure 4C). Notably, the expression of most interferon‐stimulated genes (ISGs) were maintained or even increased versus untreated PAO1 mice, indicating an interferon‐independent ISG program that may support pathogen clearance (Figure 4C). Together, these changes in cytokine and chemokine profiles indicate a shift toward a controlled inflammatory and chemotactic response that tempers excessive signaling without compromising antimicrobial defence, crucial for limiting immune‐mediated tissue injury during infection.

*Angptl4*‐ASO also attenuated fibroblast growth factor (FGF) production and fibroblast activation pathways during PAO1 infection, evidenced by the downregulation *Tgfbr3*, *Fgfr1*, and *Pak3* (Figure 4D‐E). This anti‐fibrotic signature aligns with regulation of ECM turnover and may help prevent pathological fibrosis in infected lungs. *Angptl4*‐ASO also markedly attenuated the Gap junction channel, as evidenced by the upregulation of *Gjb1*, *Gjb4*, and *Gjb5* (Figure 4E and Figure S6B, Supporting Information). Immunofluorescence staining corroborated these findings, ZO‐1 protein was upregulatd after ASO treatment, particularly in the airway epithelium, in both PAO1 and PR8 infection models (Figure S6C, Supporting Information).

Transcriptomic profiling revealed that *Angptl4*‐ASO does not perturb baseline immunity but selectively dampens infection‐induced inflammatory programs. In the PR8 influenza infection model analyzed on day 7 post‐infection, global RNA‐seq analysis showed that ASO treatment alone elicited minimal transcriptional changes relative to untreated controls (Figure S7A, Supporting Information), indicating that *Angptl4*‐ASO does not alter steady‐state immune gene expression. By contrast, the PR8‐ASO group displayed a broad attenuation of infection‐induced gene activation, with many immune‐related transcripts showing reduced amplitude compared with the PR8 group. This pattern suggests that *Angptl4*‐ASO tempers the excessive inflammatory response to viral challenge without abolishing antiviral immunity. Consistent with this interpretation, *Angptl4*‐ASO suppressed multiple pro‐inflammatory cytokine families, including interleukins, chemokines, and IFN, during PR8 infection (Figure S7B, Supporting Information). *Angptl4*‐ASO also markedly attenuated the FGF receptor signaling pathway, highlighted by reduced expression of *Tgfbr3* and *Scd4* (Figure S7C,D, Supporting Information).

Beyond canonical inflammatory modules, *Angptl4*‐ASO uniquely modulated genes involved in protein glycosylation, with infection‐specific signatures. During PAO1 challenge, glycosylation‐related genes including *St6galnac1, Cwh43*, and *Grm7* were prominently induced (Figure S6D,E, Supporting Information). A distinct, infection‐specific set, *Fktn, Abca2*, and *Slc51b*, was similarly induced during PR8 infection (Figure S7C,D, Supporting Information), indicating that *Angptl4*‐ASO modulates glycosylation pathways in a pathogen‐dependent manner.

The lung transcriptomes point to complementary mechanisms by which *Angptl4*‐ASO confers benefit: (i) strengthening epithelial barrier function, (ii) promoting a more controlled inflammatory response with preserved host defense, and (iii) suppressing fibroblast‐associated programs and ECM remodeling. The tight coupling between inflammatory regulation and ECM dynamics further supports the potential of *Angptl4*‐targeting ASO in mitigating progression toward pulmonary fibrosis.

### 2.4 *Angptl4*‐ASO Strengthens Pulmonary Defense Barriers Through Glycoprotein Regulation

Our transcriptomic analysis revealed that *Angptl4*‐ASO treatment enhanced biological processes related to cell adhesion, gap junctions and protein glycosylation, all essential for maintaining lung barrier integrity. Post‐translational modifications such as glycosylation of tight junction membrane proteins are known to play a crucial role in sustaining epithelial cohesion and barrier function.^[^ ^29^, ^30^, ^31^ ^]^ These findings suggested that *Angptl4*‐ASO may influence glycosylation processes vital to tight junction stability and epithelial defense.

**Figure 5:**
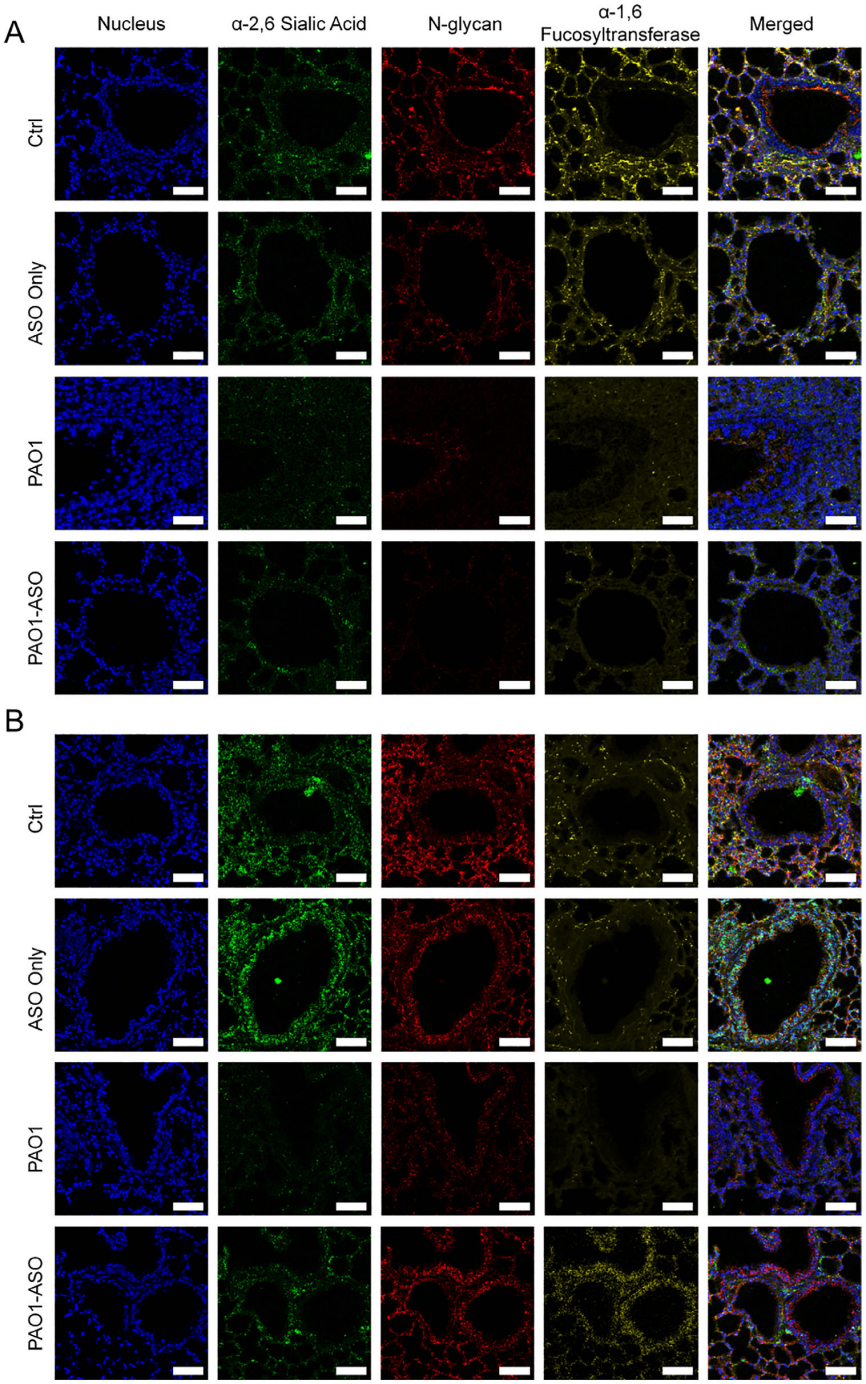
*Angptl4*‐ASO restores glycoprotein expression in infected lung tissues. Mass spectrometry imaging of lung sections showing nuclei (blue), α‐2,6 sialic acid (green), N‐glycan (red), and α‐1,6 fucosyltransferase (yellow) in Ctrl and *Angptl4*‐ASO‐only groups (top two panels). Robust expression of these glycoproteins is observed under both conditions, indicating intact pulmonary epithelial barriers. Reduced glycoprotein expression in day 2 post‐PAO1 infection A) or day 7 post‐PR8 infection B) (3^rd^ panels), reflecting damage to the epithelial barrier. In contrast, the bottom panels demonstrate that *Angptl4*‐ASO treatment restores these glycoproteins to levels comparable to those in healthy tissue, suggesting that *Angptl4*‐ASO strengthens lung barrier function through glycoprotein regulation (scale bar: 50 µm).

To test this hypothesis, we utilized mass spectrometry imaging followed by fluorescence‐based quantitative analysis to examine the distribution of glycoproteins in lung tissue (**Figure** **5**; Figure S8, Supporting Information). In healthy lungs, whether untreated or treated with ASO alone, baseline levels of key glycoproteins, including α‐2,6 sialic acid, N‐glycan, and α‐1,6 fucosyltransferase, reflected an intact pulmonary defense barrier (Figure 5, top two panels). In contrast, bacterial and viral infections significantly disrupted these glycoprotein profiles, leading to reduced expression and impaired epithelial barrier function (Figure 5, bottom two panels). Remarkably, *Angptl4*‐ASO treatment restored the levels of these glycoproteins following infection, bringing their expression closer to that observed in healthy tissue. This recovery highlights *Angptl4*‐ASO's ability to counteract infection‐induced barrier disruption by regulating glycoprotein expression.

Together, these findings suggest that *Angptl4*‐ASO strengthens pulmonary defense mechanisms by modulating glycosylation processes essential for epithelial barrier integrity. Beyond its role in inflammatory modulation, *Angptl4*‐ASO appears to safeguard infection‐ and inflammation‐driven tissue damage by strengthening molecular networks that preserve lung barrier stability and prevent fibrotic progression.

### 2.5 Targeting ANGPTL4 Attenuates Pulmonary Fibrosis Progression

Pulmonary fibrosis arises from the coupling of chronic inflammation with dysregulated tissue repair. Although ANGPTL4 modulates inflammation and tissue repair in infection settings, its precise role in pulmonary fibrosis remains unclear. We hypothesized that suppressing ANGPTL4 would attenuate fibrosis progression by limiting inflammation‐associated tissue damage.

**Figure 6:**
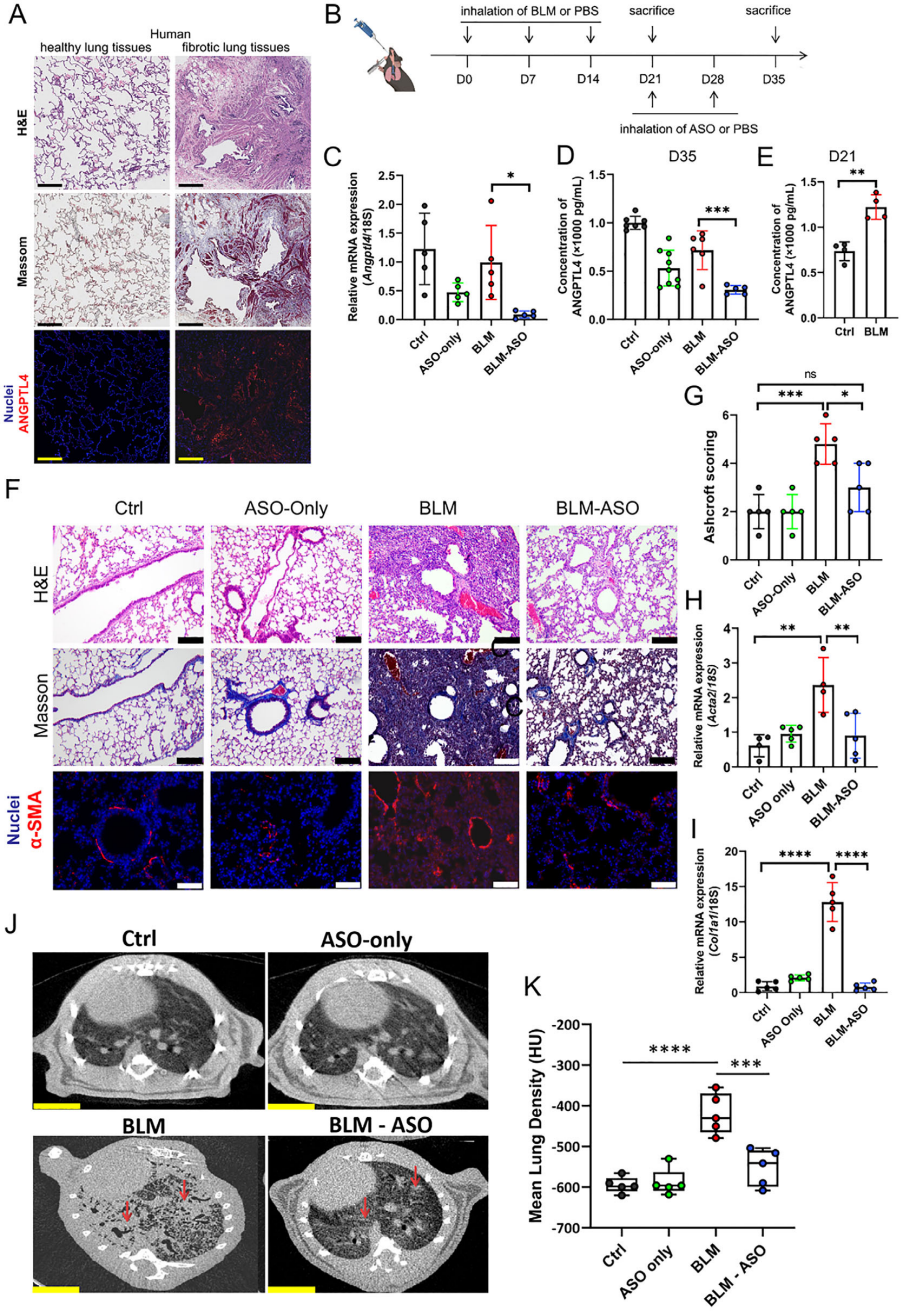
ANGPTL4 expression in human pulmonary fibrosis and treatment response in a mouse model. A) Representative images comparing normal and fibrotic human lung tissue using H&E staining (top), Masson's trichrome staining (middle), and immunofluorescence for ANGPTL4 (bottom). Normal lung tissue displays typical alveolar architecture, while fibrotic tissue shows structural disruption and elevated ANGPTL4 expression (scale bar: 500 µm). B) Experimental timeline and dosing regimen for the pulmonary fibrosis model. Schematic illustration of the experimental design for the BLM‐induced pulmonary fibrosis model. Mice received three intratracheal doses of BLM to induce lung injury and fibrosis at Days 0, 7, and 14, followed by inhaled administration of *Angptl4*‐ASO or PBS at days 21 and 28. Lung tissues and BALF were collected at days 21 and 35 for histological, biochemical, and molecular analyses. C) Relative *Angptl4* mRNA levels in mouse lung tissue across groups at day 35 post‐BLM treatment. Both ASO monotherapy and combination therapy (BLM‐ASO) significantly reduced expression compared to their respective controls. Data are shown as mean ± SD (*n* = 5 per group). Statistical comparisons were performed using one‐way ANOVA followed by Tukey’s test (*p < 0.05). D‐E. ANGPTL4 protein quantification at D) day 35 and E) day 21 post‐BLM treatment. BLM treatment increased ANGPTL4 levels at day 21, while ASO therapy significantly reduced expression at day 35. D) data are shown as mean ± SD (*n *= 5‐9 per group). Statistical comparisons were performed using one‐way ANOVA followed by Tukey’s test (***p < 0.001). E) data are shown as mean ± SD (*n *= 4 per group). Statistical comparisons were performed using two‐tailed unpaired Student's *t*‐test (**p < 0.01). F) Lung tissue analysis across groups using H&E staining (top), Masson's trichrome staining (middle), and α‐SMA immunofluorescence (bottom) at day 35 post‐BLM treatment. Ctrl and ASO‐only groups show normal architecture, while BLM treatment induced structural changes that were partially ameliorated by ASO therapy (scale bar: 200 µm). G‐I. Quantitative assessments showing G) Ashcroft fibrosis scoring, H) *Acta2* mRNA expression, and I) *Col1a1* mRNA expression across groups at day 35 post BLM‐treatment. Data are shown as mean ± SD (*n* = 4 or 5 per group). Statistical comparisons were performed using one‐way ANOVA followed by Tukey’s test (*p < 0.05, **p < 0.01, ***p < 0.001, ****p < 0.0001, ns, *p* > 0.05). J‐K. J) Representative chest CT images and K) corresponding mean lung density measurements (in Hounsfield Units, HU) across groups at day 35 post‐BLM treatment. BLM treatment induced visible changes in lung architecture that were partially reversed by ASO therapy (scale bar: 50 µm). Data are shown as mean ± SD (*n* = 5 per group). Statistical comparisons were performed using one‐way ANOVA followed by Tukey’s test (***p < 0.001, ****p < 0.0001).

Analysis of human lung tissue revealed substantial ANGPTL4 expression in fibrotic regions, coinciding with disrupted alveolar architecture and extensive collagen deposition (**Figure** **6**A). To test the therapeutic potential of ANGPTL4 inhibition, we eused a bleomycin (BLM)‐induced mouse model of pulmonary fibrosis (Figure 6B). *Angptl4*‐ASO reduced both ANGPTL4 mRNA and protein (Figure 6C,D). ANGPTL4 protein showed temporal variation, rising markedly by day 21 post‐BLM administration (Figure 6E) and trending toward normalization at day 35 in untreated animals (Figure 6D), suggesting dynamic regulation during disease progression. Body weight monitoring showed a beneficial treatment effect of ASO treatment, and W/D ratio measurements showed significant improvement in pulmonary edema (Figure S9A,B, Supporting Information). Histological examination revealed improved lung architecture with *Angptl4*‐ASO treatment (Figure 6F), corroborated by reduced Ashcroft scores (Figure 6G) and lower expression of fibrotic markers *Acta2* and *Col1a1* (Figure 6H,I; Figure S9C, Supporting Information). Chest computed tomography further confirmed amelioration of BLM‐induced structural abnormalities by both qualitative imaging and quantitative density measurements (Figure 6J,K). Moreover, the combination of the ASO agent with pirfenidone exhibited superior efficacy in mitigating pulmonary fibrosis compared to pirfenidone alone, suggesting a potential synergistic effect (Figure S9D, Supporting Information).

To assess inflammatory remodeling, we analyzed BALF cells by flow cytometry (Figure S9E,F, Supporting Information). Relative to untreated BLM group, *Angptl4*‐ASO significantly reduced the proportion of macrophages without altering total leukocyte counts. BALF cytokine profiling revealed CCL17, CCL20, IL‐2 in ASO‐treated mice, suggesting enhanced regulatory/repair programs that may restrain fibrogenesis (Figure S9G–I, Supporting Information).

To define shared versus model‐specific mechanisms, we performed comparative lung transcriptomics across PAO1‐induced acute injury and BLM‐induced fibrosis. *Angptl4*‐ASO modulated distinct biological processes in each model, epithelial morphogenesis, circulatory regulation, proteolysis, humoral responses, and ECM organization in BLM; and carbohydrate/sterol metabolism, steroid hormone responses, secretory cell differentiation, and mediator release in PAO1 (Figure S10A, Supporting Information). Importantly, ≈ 45.7% (16 hallmarks) of significantly altered hallmarks were common to both lung injury models (Figure S10B, Supporting Information). These shared pathways, encompassing hypoxia, TNFα‐NFκB signaling, apoptosis, p53 response, mitotic cell cycle regulation, and sterol metabolism, indicate a conserved ANGPTL4‐regulated network that coordinates injury resolution irrespective of the initiating insult, while model‐specific programs reflect context‐dependent modulation.

These data demonstrate that inhaled *Angptl4*‐ASO attenuates both inflammatory and fibrotic components of lung injury, improving structure and function while engaging conserved repair pathways. Importantly, *Angptl4*‐KO mice exhibited comparable protection across PAO1, PR8, and BLM challenges, mirroring the effects of ASO therapy (Figure S10C, Supporting Information). In contrast to systemic administration, we adopted an inhalation delivery approach for the ASO drug in this study. To evaluate its pulmonary retention and biodistribution, the ASO was conjugated with a Cy5 fluorescent tag. Following inhalation, the fluorescence intensity was measured in the heart, liver, spleen, lung, kidney, and blood at 2, 48, 96, and 144 h (Figure S11, Supporting Information). The drug was predominantly detected in the lungs, with minimal distribution observed in the heart, liver, spleen, kidney, and blood. Notably, a substantial signal remained in the lungs even at the 144‐h time point. These results indicate that the inhaled ASO formulation achieves prolonged retention in the lungs, supporting its potential for sustained local action.

These results establish ANGPTL4 as a compelling therapeutic target in pulmonary fibrosis and support the broad applicability of ANGPTL4‐directed, lung‐targeted antisense therapy.

## 3 Discussion

Lung injury and fibrosis are significant clinical challenges characterized by rising incidence rates and limited therapeutic options. Current therapies such as corticosteroids for inflammation and antifibrotic drugs like pirfenidone and nintedanib offer modest benefit and are frequently accompanied by side effects and potential drug resistance. This underscores an urgent unmet need for innovative and targeted therapeutic strategies. ANGPTL4, a key mediator of vascular permeability, inflammation, and tissue remodeling, has emerged as a compelling therapeutic target due to its central role in lung pathology.

In this study, we introduce *ANGPTL4*‐ASO as a novel therapeutic approach, engineered with modified nucleotides and sulfate acid bonds to enhance stability, binding affinity, and target specificity. Our findings demonstrate that *ANGPTL4*‐ASO effectively reduces ANGPTL4 protein by promoting RNase‐mediated mRNA degradation, as confirmed by transcriptomic analyses. In a severe lung inflammation model induced by *P. aeruginosa*, *Angptl4*‐ASO mitigated inflammatory responses, preserved pulmonary architecture, and enhanced pathogen clearance by engaging critical immune pathways.

Further supporting its therapeutic potential, *ANGPTL4*‐ASO displayed broad‐spectrum efficacy across diverse respiratory pathogens. Our data from bacterial (*P. aeruginosa*) and viral (influenza) infection models indicate robust modulation of host immune responses, offering a host‐targeted strategy overcome limitations of conventional antimicrobials and to reduce illness severity and duration across pathogens.

Comparative transcriptomics clarified both shared and model‐specific responses to *Angptl4*‐ASO treatment across acute and chronic lung injury. Distinct signatures such as heightened immune and oxidative stress pathways in PAO1, and enrichment of ECM remodeling and TGFβ/Wnt signaling in BLM, underscore context‐dependent modulation following ANGPTL4 suppression. Notably, ≈45.7% of significantly modulated hallmarks, including hypoxia, TNFα‐NFκB signaling, apoptosis, p53‐mediated responses, mitotic cell cycle regulation, and sterol metabolism, were shared, defining a conserved ANGPTL4‐regulated network that promotes injury resolution irrespective of the initiating insult. This overlap highlights ANGPTL4 as a master regulator in pulmonary pathophysiology and supports *ANGPTL* *4*‐ASO's versatile applicability.

Tissue leakage is a hallmark of severe infections, such as dengue virus and SARS coronavirus.^[^ ^32^ ^]^ During lung infections, cytokine‐driven increases in vascular permeability recruit immune cells and antibodies to the site.^[^ ^33^ ^]^ In severe cases, hypercytokinemia triggers uncontrolled inflammation, leading to tissue damage.^[^ ^34^, ^35^ ^]^ ANGPTL4 exacerbates this by disrupting vascular endothelial integrity, interfering with integrin signaling, and destabilizing VE‐cadherin and claudin‐5.^[^ ^36^ ^]^ Beyond modulating these interactions, *Angptl4*‐ASO reinforced pulmonary defense barriers through glycoprotein regulation. Specifically, treatment increased α‐2,6 sialic acid, N‐glycan, and α‐1,6 fucosyltransferase levels in mouse lungs. Glycosylation of tight junction proteins emerged as a pivotal post‐translational modification underpinning epithelial barrier stability, adding a new dimension to ANGPTL4's mechanism of action.^[^ ^37^, ^38^ ^]^ Unlike corticosteroids, which can worsen vascular leakage and carry systemic risks,^[^ ^39^, ^40^, ^41^, ^42^ ^]^ *Angptl4*‐ASO modulates host immunity and glycoprotein regulation, offering a potentially safer and more effective approach to managing lung inflammation.

Pulmonary fibrosis, marked by high mortality and disability, is increasing with aging population and environmental stressors. Although etiologies are diverse, many converge on chronic inflammation and aberrant repair. In our fibrosis model, *Angptl4*‐ASO reduced macrophage proportion in BALF and increased CCL17, CCL20, and IL‐2 in lung tissues. Prior studies show IL‐2 enhances Treg cell function to reverse tissue damage and inflammation,^[^ ^43^ ^]^ and to promote repair while suppressing fibrosis.^[^ ^44^, ^45^, ^46^ ^]^ Low‐dose IL‐2 can alleviate pulmonary fibrosis and vascular remodeling in mice.^[^ ^47^ ^]^ Previous research show both CCL17^[^ ^48^, ^49^, ^50^, ^51^ ^]^ and CCL20^[^ ^52^, ^53^, ^54^ ^]^ can recruit Treg cells in mice. Thus, IL‐2, CCL17, and CCL20 increases after ASO treatment likely contribute to antifibrotic activity by expanding and activating Tregs to modulate immune tone. Consistent with this, *Angptl4*‐ASO reduced collagen deposition and α‐SMA more effectively than standard antifibrotics in bleomycin‐induced disease, addressing fibrosis via pathways integral to resolution and regeneration rather than merely slowing decline.

Inhaled delivery further enhances clinical potential by localizing activity to lung tissue, minimizing systemic exposure, and improving patient compliance.^[^ ^55^ ^]^ This strategy leverages the precision of antisense technology while directly addressing a key translational challenge, selective, efficient tissue delivery. Nevertheless, limitations remain. Comprehensive pharmacokinetic and biodistribution studies are ongoing to define relationships among lung retention, plasma exposure, and pharmacodynamic response after inhalation. Long‐term safety and repeat‐dose tolerability also require further evaluation. These will be addressed in the IND‐enabling work through route‐specific PK/PD modeling and inhalation toxicology. From a mechanistic standpoint, glycosylation is a complex and heterogeneous, the precise pathways by which *ANGPTL4*‐ASO modulates epithelial glycoprotein regulation and barrier restoration remain to be fully elucidated. Follow‐up glycoproteomic and lectin‐based studies will be required to define these interactions in greater molecular detail.

In conclusion, *ANGPTL4*‐ASO is a promising therapeutic strategy for lung injury and fibrosis. By modulating immune responses, reinforcing tissue integrity, and attenuating fibrosis progression, and by engaging a conserved repair programs across distinct insults, *ANGPTL4*‐ASO addresses key pathological hallmarks across diverse contexts. These properties support its potential to improve clinical outcome and broaden therapeutic options in respiratory medicine.

## 4 Materials and Methods

### 4.1 Cell Culture and *Angptl4*‐ASO/*ANGPTL4*‐ASO Test

The human epidermal keratinocyte, HaCaT and mouse healthy hepatocyte, AML12 were obtained from American Type Culture Collection (ATCC; VA, USA) cultured at 37 °C, 95% humidity and 5% CO_2_. HaCaT were maintained in DMEM supplemented with 10% fetal bovine serum (FBS; A5256701, Gibco, MA, USA) while AML12 were maintained in advanced DMEM/F12 (12634010, Gibco, MA, USA) supplemented with 10% FBS, 1x insulin‐transferrin‐selenium (ITS; 41400045, Gibco, MA, USA), and 40 ng/mL dexamethasone (D4902, Sigma‐Aldrich, Darmstadt, Germany).

To examine the efficacy of *Angptl4*‐ASOs, the cells were cultured in a 6‐well plate until 70% confluency. The cells were then prepared for the ASO transfection by switching the culture medium to Opti‐MEM (31985070, Gibco, MA, USA). To induce *ANGPTL4* overexpression, the cells were incubated in a hypoxic chamber at < 1% O_2_ for 12 h and cotreated with varying concentrations of *ANGPTL4*‐ASOs. IC50 values were calculated from 6‐point titration curves ranging from 1 nM to 100 µM.

### 4.2 Animals and *Angptl4*‐ASO

For pulmonary infection mouse model, female C57BL/6J mice (8 to 12 weeks old) were purchased from Beijing Vital River Laboratory Animal Technology Co., Ltd. and kept at the biosafety level‐2 (BSL‐2) animal facility of the Shenzhen Institute of Advanced Technology or Shenzhen Center for Disease Control and Prevention. All animal experiments were approved by the Institutional Animal Care and Use Committee (IACUC) at Shenzhen Institute of Advanced Technology (SIAT‐IACUC‐200818‐YYS‐LL‐A1049‐02), and Shenzhen Center for Disease Control and Prevention (2025039). For pulmonary fibrosis mouse model, Male C57BL/6 (12 weeks old) were purchased from Beijing Vital River Laboratory Animal Technology Co.,Ltd. and housed in a Specific Pathogen Free (SPF) animal facility of Southern University of Science and Technology. The animal studies were approved and performed in accordance with guidelines established by the Animal Ethics Committee of Southern University of Science and Technology (No. SUSTech‐JY202209010). In both infectious and fibrosis model, *Angptl4* Knockout (KO) mice, kindly provided by the laboratory of Prof. Nguan Soon Tan, were bred to homozygosity and used alongside their wild‐type counterparts.

### 4.3 Patient Samples

Lung tissues of the fibrotic and associated healthy regions were collected from the surplus materials of surgically removed lung tissues from patients diagnosed with pulmonary fibrosis. This study was approved by the Institutional Review Board of the Seventh Affiliated Hospital of Sun Yat‐sen University (IRB Approval Number KY‐2024‐304‐02), and informed consent was obtained from all participants.

### 4.4 Culture of PAO1

Frozen bacterial strains from the −80 °C refrigerator and allow them to thaw slightly. 200 µL of the bacterial solution cultured in Luria–Bertani (LB; L8291, Solarbio, Beijing, China) medium at rotational speed to 150 rpm and the temperature to 37 °C. Prior to inoculation, the bacterial solution was diluted to a turbidity of 2.5 using Massey's turbidimetry, which corresponds to a bacterial concentration of 10^7^ CFU/mL.

### 4.5 Influenza Virus

Influenza virus A/Puerto Rico/8/34 H1N1 strain (PR8) was obtained from the American Type Culture Collection. The virus was propagated in embryonated eggs and plaque assay was applied to assess the viral titers.

### 4.6 Construction of Mouse Models Infected with *P. aeruginosa* or Influenza PR8 Virus

Mouse models of pulmonary infection caused by *P. aeruginosa* and influenza PR8 virus were established as previously described.^[^ ^11^, ^56^, ^57^ ^]^ For *P. aeruginosa* infection, mice were anesthetized with 2,2,2‐tribromoethanol (T48402, Sigma‐Aldrich, Darmstadt, Germany) by intraperitoneal injection and infected intratracheally with 1 × 10^6^ CFU of *P. aeruginosa*. In the acute infection model, mice received a single infection and were euthanized 2 days post‐infection. In the repeated infection model, mice were infected on Day 0 and Day 5 and euthanized on Day 10 after the first infection. For PR8 infection, mice were anesthetized as above and infected intratracheally with 40 PFU of PR8. In the single infection model, mice received a single infection and were euthanized 7 days post‐infection. In the repeated infection model, mice were infected on Day 0 and Day 5 and euthanized on Day 10 after the first infection. For ASO treatment, 24 µg *Angptl4*‐ASO was administered by intratracheal inhalation at the time of *P. aeruginosa* or PR8 infection. Mice receiving sterile Posphate Buffered Saline (PBS; C10010500BT, Gibco, MA, USA) or the same ASO without infection served as blank and ASO controls, respectively. At the indicated time points, lungs, bronchi, liver, and kidneys were harvested for downstream analyses. The left upper lung was used for bacterial load measurement. The right upper lung was fixed in 4 % PFA solution for subsequent HE and immunofluorescence staining. The remaining lung lobes were used for RNA sequencing and qPCR.

### 4.7 Dose‐Response Curve and qPCR

To determine the dose response and IC50 of each ASO, cells were exposed hypoxic condition and cotreated with increasing concentrations of ASOs (1 to 1 × 10^5^ nM) for 12 h. After the treatment, the cells were lysed for total RNA extraction. Expression of *ANGPTL4* was evaluated using qPCR and normalized to TATA‐box binding protein gene (*TBP*). The primers used are as follows. IC50 values of the ASOs were determined using GraphPad Prism (GraphPad Software Inc., CA, USA) by fitting the fold change values to a non‐linear regression (**Table** **1**).

**Table 1:** Nucleotide sequences of qPCR primers.

Gene	Species	Forward primer (5′ to 3′)	Reverse primer (5′ to 3′)
*ANGPTL4*	Human	TGG TTT GGC ACC TGC AGC CAT TC	TGC TGC CAT GGG CTG GAT CAA C
*TBP*	Human	GCT GGT TAT CGG GAG TTG G	GGG AGG CAA GGG TAC ATG AG
*Angptl4*	Mouse	CCC CAC GCA CCT AGA CAA TG	GCC TCC ATC TGA AGT CAT CTC A
18S	Mouse	GTA ACC CGT TGA ACC CCA TT	CCA TCC AAT CGG TAG TAG GG
*Acta2*	Mouse	CCC AGA CAT CAG GGA GTA ATG G	TCT ATC GGA TAC TTC AGC GTC A
*Col1a1*	Mouse	CCA AGA AGA CAT CCC TGA AGT CA	TGC ACG TCA TCG CAC ACA

### 4.8 Inflammatory Cell Count

Three mice were randomly selected from each experimental group, and 10 randomly selected 40× microscopic fields were analyzed per mouse using hematoxylin and eosin staining. The number of inflammatory cells within the fields was counted for statistical analysis.

### 4.9 Bacterial Load Analysis

Lung tissue was weighed and then soaked in 500 µL of sterile PBS. The tissue was fragmented using a handheld ultrasound tissue crusher (15‐345‐136, Branson, CT, USA) until no obvious tissue blocks remained. A tissue suspension was prepared and diluted 10‐, 100‐ and 1000‐fold. Next, 3 µL of each dilution were taken and plated in triplicate onto LB agar. The culture plates were incubated at 37 °C for 12 h, and the number of bacterial colonies was counted to determine the bacterial load per unit weight of lung tissue.

### 4.10 Assessment of Health Status

We developed a composite health score (**Table** **2**) that integrates data from body weight loss, physical condition, and activity level, providing a more robust and multidimensional evaluation under these acute experimental conditions.

**Table 2:** Composite health scoring criterion.

Parameter	0 (Severe Abnormality)	1 (Moderate Abnormality)	2 (Normal/Healthy)
Body Weight Loss	>20%	10%–20%	<10%
Physical Condition	Severe emaciation, bones highly prominent	Mild wasting, bones easily palpable	Normal conformation, well‐fleshed
Activity Level	Immobile or only respiratory movements	Reduced activity, sluggish response to stimuli	Normal exploration and activity
Posture/Hunching	Persistent severe hunching, curled posture	Intermittent mild hunching	Normal, relaxed posture
Coat Condition	Severely ruffled, piloerected, dull	Mild localized ruffling, less glossy	Coat smooth, flat, and glossy
Respiration	Labored breathing, abdominal respiration	Slightly rapid or abnormal breathing	Smooth and normal
Response to Stimulus	No response	Sluggish response	Normal response (escape, grooming, alertness)
Eyes	Deeply sunken, cloudy, significant discharge	Mildly sunken or minimal discharge	Bright, fully open, no discharge
Dehydration	Skin tent > 2 seconds	Slow skin tent (1‐2 seconds)	Rapid skin recoil (<1 second)
Spontaneous Behavior	No grooming, no nesting	Markedly reduced behaviors	Normal grooming, nesting, foraging
For each parameter, the score ranges as 0, 1, and 2. The maximum total score for all the 10 parameters for each mouse is 20. Individual score is thus calculated for each mouse and recorded below.			
Individual Score:			
Health Status ((Subtotal Score/20)*100):			

### 4.11 Measurement of Lung Wet/Dry Weight Ratio

The left lower lobe of the lung was carefully dissected and immediately weighed to obtain the wet weight. The lobe was then placed in a drying oven at 60 °C for 48 h and weighed again to obtain the constant dry weight. The lung W/D ratio was calculated as follows: W/D ratio = Wet Weight / Dry Weight.

### 4.12 Mass Spectrometry Imaging

The mass spectrometry imaging was performed as published previously.^[^ ^58^ ^]^ Formalin‐fixed, paraffin‐embedded (FFPE) lung tissue sections were deparaffinized and rehydrated. Antigen retrieval was performed in a retrieval buffer (G1202, Servicebio, Wuhan, China) at 96 °C for 30 min, followed by natural cooling to room temperature. Slides were washed with Dulbecco’s phosphate‐buffered saline (DPBS; C14190500BT, Gibco, MA, USA) and then blocked with 5% goat serum (PH0424, Phygene Biotechnology, Fuzhou, China) in DPBS for 45 min at room temperature. After blocking, sections were incubated with a primary antibody cocktail at 4°C overnight. The following day, slides were washed with 0.2% Triton X‐100 (abs9149, Absin, Shanghai,China) in DPBS, incubated with metal‐tagged secondary antibodies for 1 h at room temperature, and then washed with 0.1% Triton X‐100 in DPBS. Sections were stained with an Ir‐Intercalator for 30 min at room temperature, rinsed with ddH_2_O, and air‐dried for over 20 min. Imaging was performed using an imaging mass cytometer (Fluidigm Hyperion, Standard BioTools Inc., MA, USA). Quantitative fluorescence analysis was performed by ImageJ (version: 1.8.0_66; NIH, MD, USA).

### 4.13 qPCR

Primers were synthesized by Sangon Biotech (Shanghai, China) using published murine sequences from GenBank (see Table 1). Total RNA was extracted using TRIzol (15596018CN, Thermo Fisher Scientific, MA, USA) and reverse transcribed with the PrimeScript RT reagent kit (RR036A, Takara, Japan). Quantitative PCR (qPCR) was conducted using SYBR Green‐based qPCR kit (QPS‐201, TOYOBO, Osaka, Japan), with each reaction performed in triplicate. Relative gene expression changes were normalized to the housekeeping gene *TBP* or 18S and calculated using the 2−ΔΔCT method.

### 4.14 ELISA Measurement of ANGPTL4 Protein and Cytokines

Proteins were extracted from mouse lung tissue and their concentrations were determined using the Bicinchoninic Acid Assay (BCA; Thermo Fisher Scientific, MA, USA). The levels of ANGPTL4 were measured according to the manufacturer's instructions for the Mouse ANGPTL4 ELISA Kit (Ab210577, Abcam, Cambridge, UK). The levels of cytokines were measured according to the manufacturer's instructions for the Bio‐Plex Pro Mouse Chemokine 31‐Plex kit (12009159, Bio‐Rad, CA, USA).

### 4.15 Construction of a Bleomycin‐Induced Pulmonary Fibrosis Mouse Model

Mouse models of pulmonary fibrosis induced by bleomycin were established as previously described^[^ ^59^, ^60^ ^]^   Pulmonary fibrosis of mice was induced by intratracheal instillation of BLM (2mg/kg; 2197601, HanHui Pharmaceuticals Co., Ltd., China) once weekly for three consecutive weeks. For ASO treatment, 12 µg ASO was administered by intratracheal inhalation once weekly for two weeks after pulmonary fibrosis was established. Mice receiving sterile PBS or the same ASO without BLM treatment served as blank and ASO controls. Body weight was monitored weekly. To assess the ANGPTL4 expression level, a subset of mice were euthanized on Day 21 after the first BLM instillation. All remaining mice were enthanized on Day 35 for endpoint analysis. Additional groups involving pirfenidone (PFD; Beijing Continent Pharmaceuticals Co., Ltd., Beijing, China) treatment were conducted to assess combinatorial effects, with results presented in the Supporting Information. The left lung was collected and fixed in 4% paraformaldehyde (G1101, Servicebio, Wuhan, China) solution for histological sectioning and staining. Bronchoalveolar lavage fluid (BALF) was collected, and the remaining lung lobes were snap‐frozen for subsequent analyses.

### 4.16 Micro‐CT Imaging and Lung Density Measurement

Mouse lung CT scans were performed using micro‐CT (Bruker, MA, USA). Set the scanning parameters to 60 kV and 100 µA, and calibrate the bright and dark fields. Anesthetize the mice, secure them in the dedicated animal scanning box with cotton tape, and begin scanning. Fill an EP tube with double‐distilled water, place it in the aforementioned scanning box, and scan the pure water. Reconstruct the samples using NRecon software (version: 1.6.8.0; Bruker, MA, USA). Given that the HU value of pure water is 0 and the HU value of air is ‐1000, use these as standards to determine the attenuation coefficient with CTan software (version: 1.12.10.0+; Bruker, MA, USA). Delineate the target areas of the mouse lungs using CTan software and calculate the average HU value (density value) of the mouse lungs based on the attenuation coefficient.

### 4.17 Flow Cytometric Analysis of BALF and Lung Tissue

Bronchoalveolar lavage fluid (BALF) was collected by instilling and withdrawing ice‐cold PBS. Lung tissues were harvested and dissociated into single‐cell suspensions using enzymatic digestion. Cells from both sources were stained with fluorescently labeled antibodies against surface markers for 30 min (see **Table** **3** for the antibody panes). More details on the experimental procedures can be found in Supporting Information. Data were analyzed using Cytexpert (version: 2.6.0.105, Beckman Coulter.Inc, CA, USA) to determine immune cell population frequencies.

**Table 3:** Antibody panels for flow cytometry.

Panel 1	Target	Fluorochrome	Species	Brand	Catalog number
1	CD45	PE	Mouse	Miltenyi	130‐110‐659
2	CD3	BV421	Mouse	BD Biosciences	740 014
3	CD4	Percp‐Cy5.5	Mouse	Thermo Fisher	45‐0042‐82
4	CD8	APC	Mouse	BioLegend	100 712
5	CD19	FITC	Mouse	BioLegend	115 506
Panel 2	Target	Fluorochrome	Species	Brand	Catalog Number
1	CD45	Percp‐Cy5.5	Mouse	BioLegend	45‐0451‐82
2	Ly6g	FITC	Mouse	Thermo Fisher	11‐9668‐82
3	NK1.1	APC	Mouse	BioLegend	108 710
4	F4/80	PE	Mouse	BioLegend	123 114

### 4.18 Transcriptome Analysis

Total RNA was extracted and integrity assessed as previously described.^[^ ^25^ ^]^ Libraries were constructed using the VAHTS Universal V6 RNA‐seq Library Prep Kit according to the manufacturer's instructions, and sequencing was conducted by OE Biotech Co., Ltd. (Shanghai, China) using an Illumina NovaSeq 6000 platform to generate 150 bp paired‐end reads. Raw reads in fastq format were initially processed with fastp^[^ ^61^ ^]^ to remove low‐quality reads. Clean reads were mapped to the reference genome using HISAT2,^[^ ^62^ ^]^ and gene expression was quantified as FPKM,^[^ ^63^ ^]^ with gene counts obtained via HTSeq‐count.^[^ ^64^ ^]^ Differential gene expression analysis was performed using DESeq2^[^ ^65^ ^]^ after filtering out genes with low expression (counts < 10 in the minimum number of samples per condition group). Differentially expressed genes (DEGs) were defined by a Benjamini‐Hochberg adjusted *p*‐value < 0.05 and an absolute log_2_ fold‐change >0.585. To visualize global transcriptomic patterns and evaluate potential batch effects, principal component analysis (PCA) was performed on variance‐stabilized transformed (VST) count data using the plotPCA function within DESeq2. Volcano plots were generated to display the relationship between statistical significance and magnitude of change for all genes, with the negative base‐10 logarithm of the adjusted p‐value plotted against the log_2_ fold‐change; DEGs meeting the significance thresholds were highlighted. Venn diagrams were created using the ggVennDiagram package to illustrate the overlap of significant gene sets identified across different experimental comparisons. Hierarchical clustering of DEGs was visualized with ComplexHeatmap, and genes were partitioned into distinct clusters using k‐means clustering. Functional characterization of gene clusters was conducted through Gene Ontology Biological Process (GO:BP) over‐representation analysis using clusterProfiler. Gene Set Enrichment Analysis (GSEA) was also performed using the fgsea package on all expressed genes ranked by their DESeq2 Wald statistic, identifying significantly enriched MSigDB Hallmark and GO:BP pathways (adjusted *p*‐value < 0.05). Additionally, radar maps highlighting the top 30 DEGs and diagrams illustrating significantly enriched terms were generated using R (version: 3.2.0). Pathway analyses were further refined using Ingenuity Pathway Analysis (IPA; QIAGEN), filtering results based on *p*‐values and *z*‐scores.

### 4.19 Statistics

GraphPad Prism software (version: 8.0.2; GraphPad Software Inc., CA, USA) were used. Data were presented as indicated in the figure legends. The two‐tailed unpaired Student's *t*‐test, two‐way ANOVA followed by Tukey’s test, one‐way ANOVA followed by Tukey's test, or Kruskal‐Wallis test with Dunn's post‐hoc test were used for intergroup comparison and *p* < 0.05 was considered statistically significant.

## Conflict of Interest

All authors, except MMJ and SKN, declare no conflicts of interest. MMJ and SKN are employee of Lipigon Pharmaceuticals AB, Umeå, Sweden, but was not involved in the study design nor interpretation of the data.

## Author Contributions

F.H., T.Y., and Z.J. performed the experiments, data analysis, and contributed equally to writing the manuscript; L.X. performed the experiments and pathological analysis; D.C., H.S.C., and Y.X.L. performed in vitro cell culture experiments and data analysis; C.Q. performed the pathological scoring; Q.J., D.L., J.Q., and Y.L. performed CT scanning and data collection; M.C., M.B., Z.Q., and L.Y. contributed to clinical sample studies; M.M.J. and S.K.N. designed and synthesized the ASO and reviewed the manuscript; L.L., N.S.T., and F.Y. supervised, review and edited the manuscript; L.L. and N.S.T. designed and conceptualized the study. All authors read and approved the final manuscript.

## Supporting information



Supporting Information

## Data Availability

The data that support the findings of this study are openly available in GSE288259 at https://www.ncbi.nlm.nih.gov/geo/query/acc.cgi?acc=GSE288259, reference number 288259.
