# Supplementary material for: Inhaled Angiopoietin‐Like 4 Antisense Oligonucleotide Therapy for Lung Injury and Fibrosis
Source: Adv Sci (Weinh). 2026 Mar 23;13(23):e01909. doi: 10.1002/advs.202501909 (PMC13104087; doi:10.1002/advs.202501909)
Supplement: Supplementary file 1 — Supporting Information [file ADVS-13-e01909-s001.docx]

**Supporting Information**

**Inhaled Angiopoietin-Like 4 Antisense Oligonucleotide Therapy for Lung Injury and Fibrosis**

**Haiyang Fan, Yuanyang Tan, Junhang Zhang, Xiaoya Liu, Jing Qu, Damien Chua, Hong Sheng Cheng, Joseph Han Sol Kim, Yu Xuan Liu, Changfei Qin, Yingzi Liu, Dezhi Li, Jikang Qiu, Mengshi Chi, Mingmin Bi, Qiwei Zhang, Yun Li, Masoumeh Motamedi Joibari, Stefan K Nilsson, Nguan Soon Tan, Yunping Fan, Liang Li**

**Supplemental Experimental Procedures**

**Combination Therapy with Pirfenidone in Bleomycin (BLM)-Induced Lung Fibrosis Model.** Pulmonary fibrosis was induced in mice by inhaled instillation of BLM (2mg/kg; 2197601, HanHui Pharmaceuticals Co., Ltd., China) once weekly for three consecutive weeks. After fibrosis establishment, therapeutic interventions began on Day 21. The pirfenidone (PFD; Beijing Continent Pharmaceuticals Co., Ltd., Beijing, China) monotherapy group received 50 mg/kg PFD daily via oral gavage from Day 21 to Day 34. The combination therapy group received the same regimen of PFD (50 mg/kg/day, Days 21–34) along with ASO (administered by inhalation on Days 21 and 28).

**Bronchoalveolar Lavage Fluid (BALF) Collection.** After anesthesia and blood collection, the left main bronchus was ligated, and the right lung was slowly lavaged twice with 0.5 mL of PBS per wash. The collected BALF was centrifuged at 400 g for 5 min at 4℃. The supernatant was aliquoted and stored at -80℃ until use. The cell pellet was incubated with 1 mL of red blood cell lysis buffer (R1010, Solarbio, Beijing, China) for 5 min at 4℃. After lysis, 10 mL of PBS was added to dilute the buffer, and the mixture was centrifuged at 400 g for 5 min at 4℃. Finally, the cell pellet was resuspended in 100 μL 1x PBS containing 1% FBS for subsequent flow cytometric staining.

**Preparation of Lung Single-Cell Suspension.** The left lung lobe was cut into small pieces and then digested in 1.5 mL of collagenase IV (0.5 mg; 17104019, Gibco, MA, USA) and dispase II (0.5 mg; 4942078001, Gibco, MA, USA) solution for 30 min at 37℃. 10% FBS DMEM medium (C11995500BT, Gibco, MA, USA) was added to stop the digestion, and the mixture was filtered through 100-μm cell strainers. The cell suspension was centrifuged at 300 g for 10 min at 4℃. The cell pellet was resuspended in 100 μL of basic DMEM, and 1mL of 1x red blood cell lysis buffer was added for a 5 min incubation on ice. After lysis, the suspension was centrifuged at 300 g for 5 min at 4℃. The final cell pellet was resuspended in 100 μL 1x PBS containing 1% FBS for subsequent flow cytometric staining.

**Histopathological Staining.** The left lung lobes of mice and the clinical lung samples were fixed with 4% paraformaldehyde (G1101, Servicebio, Wuhan, China) and embedded in paraffin. Paraffin sections were cut at a thickness of 4-5 μm. The sections were de-waxed in xylene and rehydrated in descending ethanol solution to distilled water, and then were stained with haematoxylin and eosin staining kit (H&E; PH0516, Phygene, Fujian, China) or Masson’s trichrome staining kit (G1340, Solarbio, Beijing, China). All procedures were followed according to the manufacturer’s instructions.

**Inflammatory Cell Count of Lung Sections.** For each mouse, ten randomly selected microscopic fields (40x magnification) from H&E-stained sections were analyzed. Within these fields, neutrophils and macrophages were quantified for statistical analysis based on standard morphological criteria. Neutrophils were identified by their characteristic segmented nuclei and pale eosinophilic (pink) cytoplasm. Macrophages were distinguished by their larger size, foamy cytoplasm, and vesicular nuclei that were typically round or kidney-shaped.

**Ashcroft Scoring.** Pulmonary fibrosis was assessed on Masson's trichrome-stained lung sections using the Ashcroft scoring system^1^. Five randomly chosen fields per section were examined at x100 magnification and graded on a scale of 0 (normal) to 8 (complete fibrous obliteration). The scoring criteria were as follows: Grade 0 = normal lung; Grade 1 = minimal fibrous thickening of alveolar or bronchiolar walls; Grade 3 = moderate thickening of the walls without obvious damage to lung architecture; Grade 5 = increased fibrosis with definite damage to lung structure and formation of fibrous bands or small fibrous masses; Grade 7 = severe distortion of structure and large fibrous areas; Grade 8 = total fibrous obliteration of the field. The mean score per sample was calculated and subjected to statistical analysis.

**Immunofluorescent Staining.** Lung sections were deparaffinized and heated in 1x Tris-EDTA antigen retrieval solution for 20 minutes (mouse sample) or 10 minutes (human sample sections) at 95℃, followed by blocking in solution with 5% goat serum and 2% BSA. After washing in PBS, the sections were incubated overnight at 4℃ with diluted primary antibody against ANGPTL4(1:50 dilution; in-house, Sinobiological, Beijing, China), ZO-1 tight junction (1:100 dilution; ab221547, Abcam, Cambridge, UK), and alpha-smooth muscle actin (α-SMA, 1:1000 diluted; A2547, Sigma-Aldrich, Darmstadt, Germany) in blocking buffer. After washing in 1x TBST three times, the sections were incubated with Alexa Fluor 594-labelled goat anti-rabbit secondary antibody (1:200; ab221547, Abcam, Cambridge, UK) for 1 h at room temperature, then stained with DAPI for 15 min. Finally, the images were acquired using the Whole Slide Scanner System (GScan-120Pro, G cell technology, Guangzhou, China) or Leica DMi8 Fluorescence microscope (Leica, Wetzlar, Germany). For fluorescence intensity analysis, five random fields of view (FOV) per mouse were converted to grayscale in ImageJ (version 1.8.0_66; NIH, MD, USA), and the mean fluorescence intensity was calculated.

**ANGPTL4 Enzyme-linked Immunosorbent Assay (ELISA) Measurement.** Mouse lung protein was extracted using RIPA buffer (89900, Thermo Fisher Scientific, MA, USA) supplemented with protease and phosphatase inhibitors (P1045, Beyotime, Shanghai, China), and the concentration was measured by Bicinchoninic Acid (BCA) assay kit (23227, Thermo Fisher Scientific, MA, USA). The levels of ANGPTL4 in lung samples were measured using Mouse ANGPTL4 ELISA Kit (Ab210577, Abcam, Cambridge, UK) according to the manufacturer’s instructions. For the assay, 50 μg of total protein from each sample was used.

**Bio-plex Pro Mouse Chemokine Aaaay.** To detect cytokine and chemokine concentrations in BALF supernatant, a magnetic bead-based multiplex immunoassay was performed. The Bio-Plex Assay (Bio-Plex Pro Mouse Chemokine Panel 31-Plex; 12009159, Bio-Rad, CA, USA) was performed on a Bio-Plex 200 System with a Bio-Plex Pro Wash Station. By using the median of the fluorescence intensity and the standard curve, the absolute concentration of the cytokines (CCL17, CCL20, IL2, CCL4, CCL5, CCL7, CCL25, IL6) was calculated (Bio-Plex Manager 6.1, Bio-Rad Laboratories).

**Flow Cytometric Staining.** Cells isolated from BALF or lung tissues were resuspended in 100 μL of PBS containing 1% FBS. To prevent nonspecific binding, cells were incubated with an Fc receptor blocking solution (anti-mouse CD16/32, 1:50 dilution; 553141, BD Biosciences, NJ, USA) on ice for 15 minutes. Subsequently, cells were stained with two separate antibody panels (each antibody at 1:100 dilution) for surface markers at room temperature in the dark for 30 minutes. After staining, cells were washed with PBS, filtered through a 70-μm cell strainer, and centrifuged at 400 g for 5 minutes at 4 °C. The cell pellet was then resuspended in PBS with 1% FBS. Finally, a viability dye (DAPI) was added to distinguish live/dead cells immediately before acquisition. Samples were analyzed using a flow cytometer (CytoFLEX-S, Beckman Coulter, Inc., CA, USA), and the data were analyzed using Cytexpert (version: 2.6.0.105, Beckman Coulter, Inc., CA, USA) to determine immune cell population frequencies.

***In vivo* Imaging.** Cy5-labeled ASO was synthesized (GenScript, Nanjing, China) and administered to mice via inhaled instillation under anesthesia. Prior to imaging, the mice's abdominal hair was removed to minimize background interference. Fluorescent images were acquired at 0, 2, 48, 96, and 144 hours post-administration using the AniView100 Pro Multi Mode In Vivo Imaging System (Guangzhou Biolight Biotechnology Co., Ltd., Guangzhou, China), with image quantification and analysis performed via the accompanying AniView Pro software (Version: 101902).

**Supplemental Reference**

1. Ashcroft, T., Simpson, J.M., and Timbrell, V. (1988). Simple method of estimating the severity of pulmonary fibrosis on a numerical scale. J Clin Pathol *41*, 467-470. 10.1136/jcp.41.4.467.

**
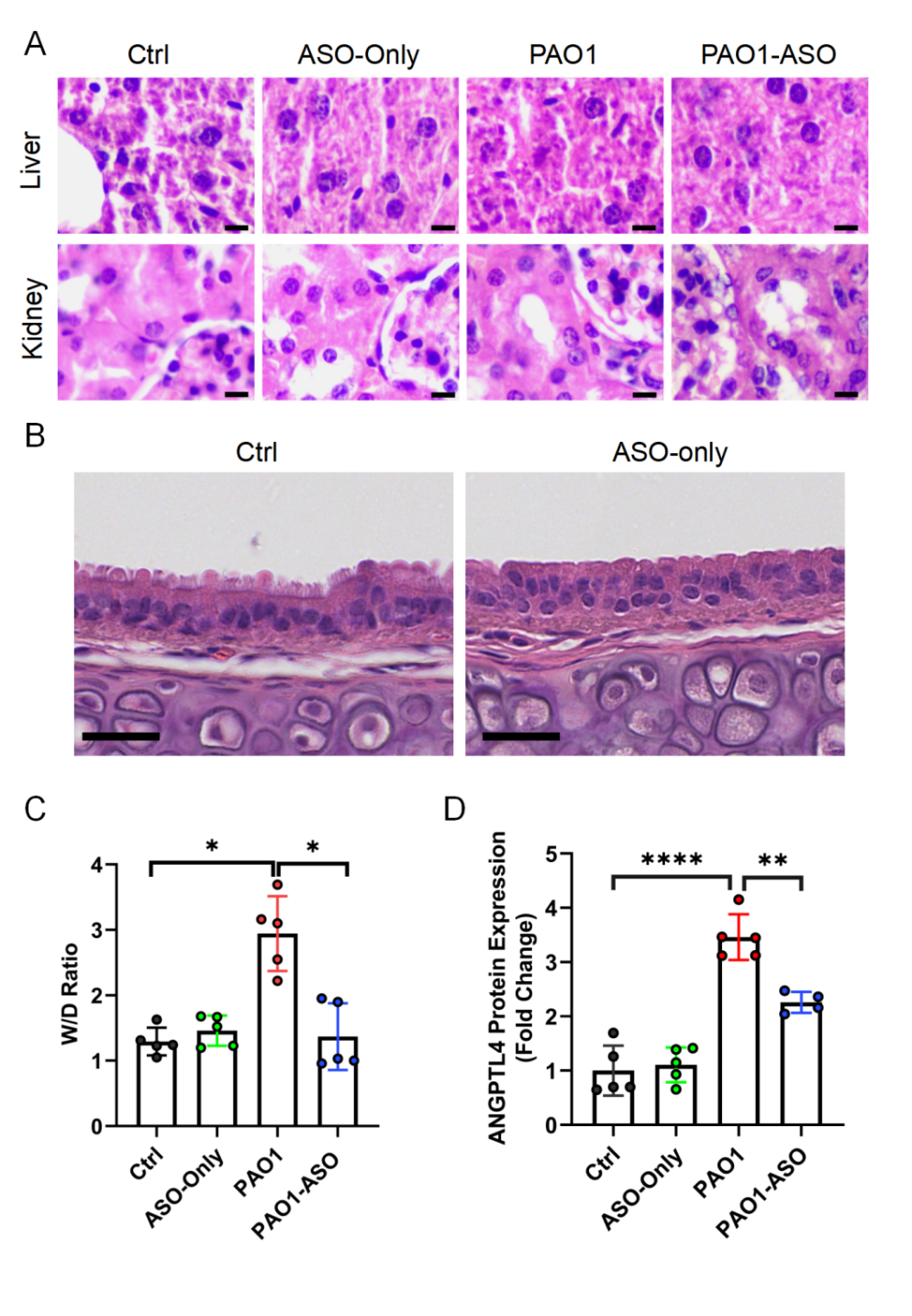
**

**Figure S1. In the acute PAO1 infection model analyzed on day 2 post-infection, inhaled *Angptl4*-ASO suppresses ANGPTL4 protein expression, reduces PAO1-induced lung edema, and does not induce detectable off-target toxicity.**

**A.** Representative hematoxylin and eosin (H&E) staining of liver and kidney sections from control (Ctrl), ASO-treated (ASO-only), PAO1-infected (PAO1), and the PAO1-infected and ASO-treated group (PAO1-ASO) groups. No histopathological abnormalities, including cellular degeneration, inflammatory infiltration, or structural disruption, were observed in ASO-treated mice compared with controls (scale bar: 10 µm).

**B.** Representative H&E-stained tracheal sections from Ctrl and ASO-only mice. Tracheal epithelium shows intact epithelial layering and no evidence of inflammation or edema, indicating that inhaled *Angptl4*-ASO administration does not induce local airway injury (scale bar: 25 µm).

**C.** Quantification of the wet-to-dry (W/D) lung weight ratio. PAO1 infection significantly increased lung edema, whereas *Angptl4*-ASO treatment markedly attenuated this response. Data represent mean ± SD (*n* = 5 per group). Statistical comparisons were performed using the Kruskal-Wallis test with Dunn’s post-hoc test (**p* < 0.05)

**D.** Fold-change in ANGPTL4 protein expression across experimental groups. PAO1 infection induced robust ANGPTL4 upregulation, which was substantially reduced following *Angptl4*-ASO administration.

Data represent mean ± SD (*n* = 4–5 per group). Statistical comparisons were one-way ANOVA followed by Tukey’s test (***p* < 0.01, *****p* < 0.0001).

**
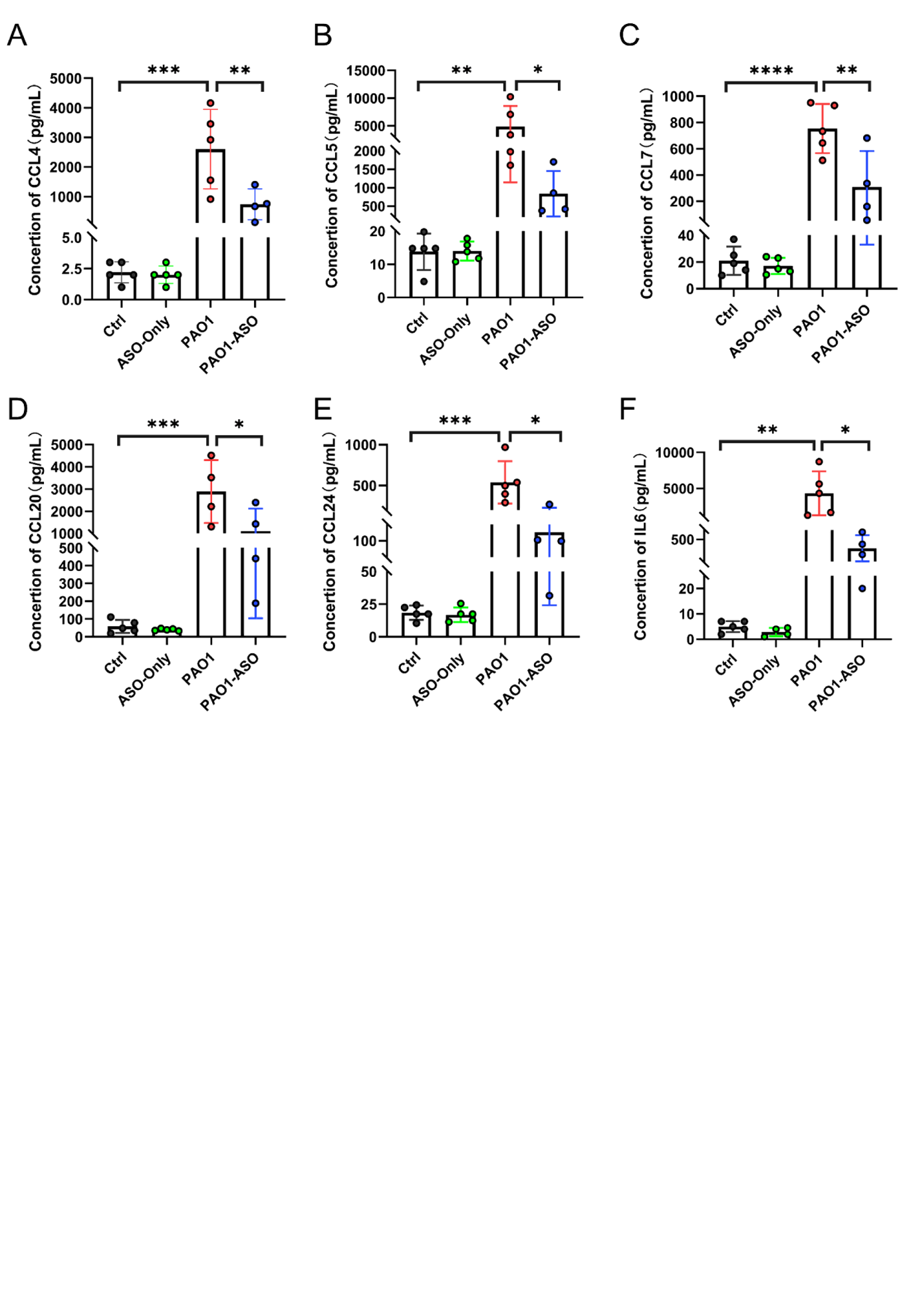
**

**Figure S2. In the PAO1 acute infection model analyzed on day 2 post-infection, ASO treatment attenuates pulmonary inflammation.**

**A-F.** In the PAO1 acute infection model, ASO treatment significantly reduced the levels of pro-inflammatory cytokines and chemokines, including CCL4, CCL5, CCL7, CCL20, CCL24, and IL-6, in bronchoalveolar lavage fluid (BALF). Data are presented as mean ± SD (*n* = 4–5 per group). Statistical significance was determined by one-way ANOVA followed by Tukey’s test (**p* < 0.05, ***p* < 0.01, ****p* < 0.001, *****p* < 0.0001).

**
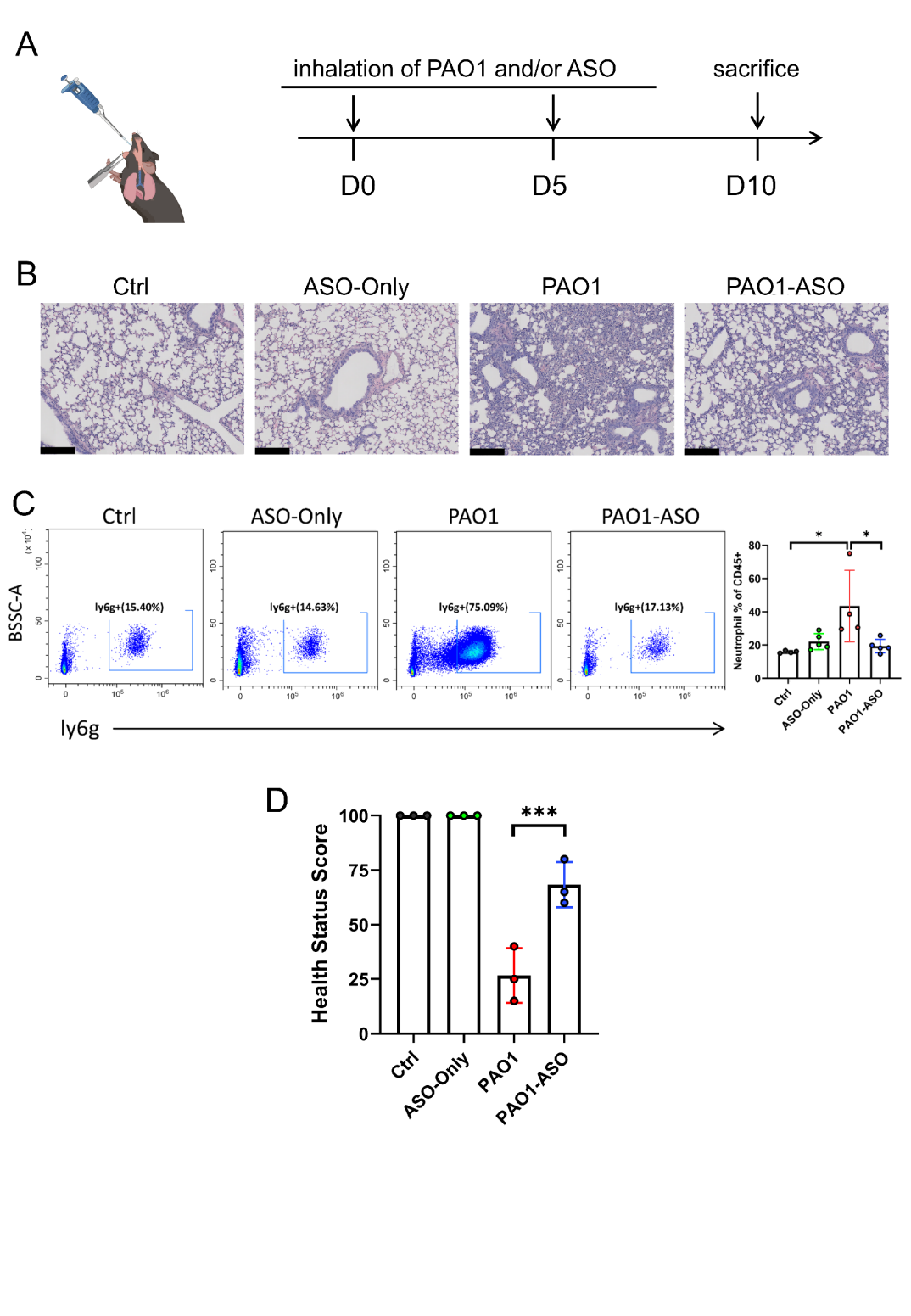
**

**Figure S3. Repeated inhaled delivery of *Angptl4*-ASO reduces lung inflammation and improves health status for mice that received repeated PAO1 infection.**

**A.** Experimental timeline of the PAO1 repeated infection model. Mice received inhaled PAO1 and/or *Angptl4*-ASO on Day 0 and Day 5, followed by sacrifice and tissue collection on Day 10.

**B.** Representative H&E staining of lung sections. Ctrl and ASO-only groups exhibited intact alveolar structure without inflammatory exudates or cellular infiltration. PAO1 infection led to extensive accumulation of inflammatory cells, alveolar wall thickening, and marked structural disruption. PAO1-ASO treatment preserved alveolar integrity and substantially reduced inflammatory infiltration (scale bar: 100 μm).

**C.** Flow cytometric analysis of lung single-cell suspensions. PAO1 infection resulted in a strong expansion of Ly6G⁺ neutrophils within the CD45⁺ compartment. *Angptl4*-ASO administration significantly reduced neutrophil abundance toward baseline levels. Data are shown as mean ± SD (*n* = 4–5 per group). Statistical comparisons were performed using one-way ANOVA followed by Tukey’s test (**p* < 0.05).

**D.** Composite health status score assessed on Day 10. PAO1-infected mice displayed pronounced clinical deterioration, whereas PAO1-ASO mice showed markedly improved health scores, indicating substantial mitigation of disease severity. Data are presented as mean ± SD (*n* = 3 per group). Statistical comparison between PAO1 and PAO1-ASO group was performed using two-tailed unpaired Student’s *t*-test (****p* < 0.001).


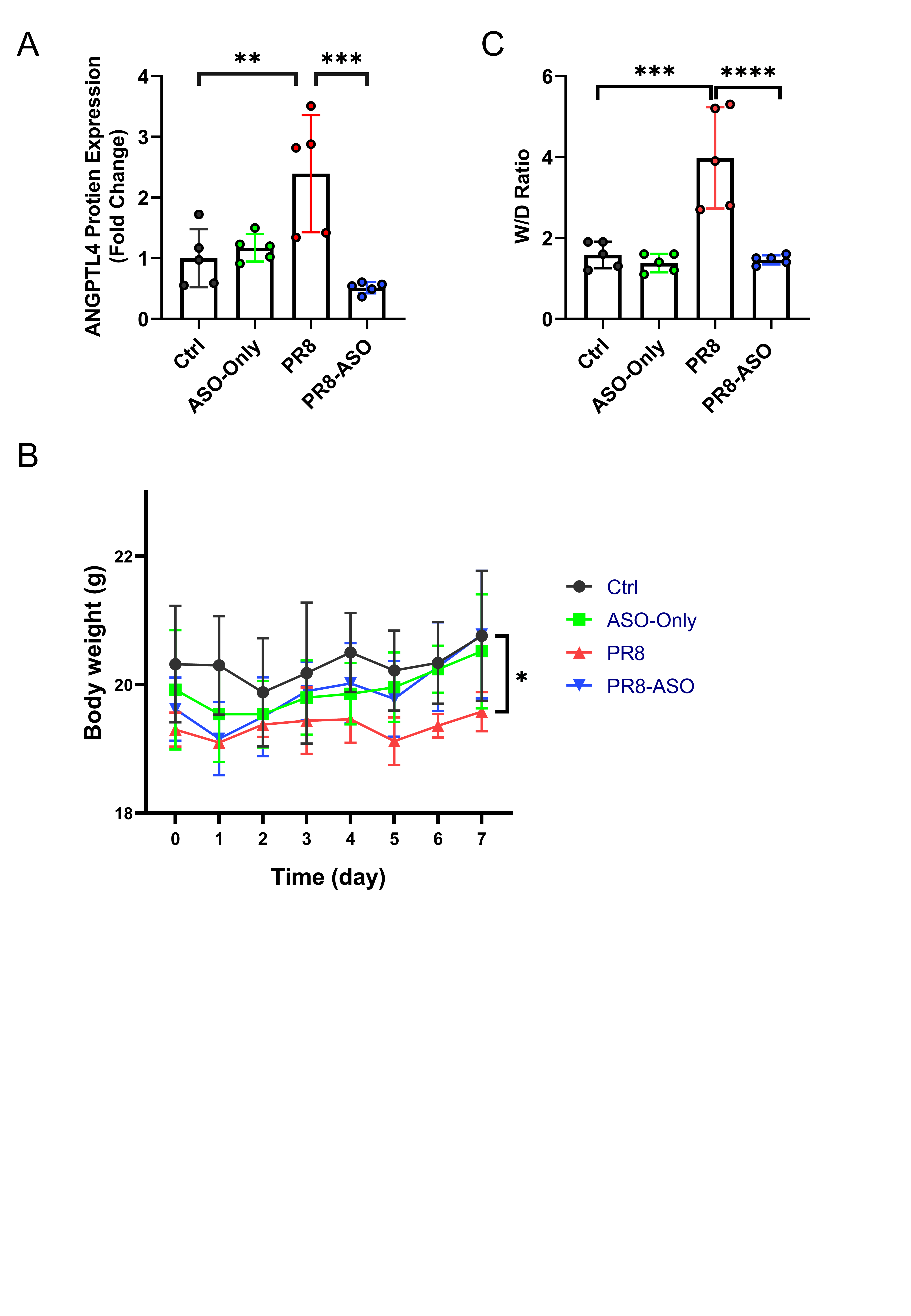


**Figure S4. *Angptl4*-ASO suppresses PR8-induced ANGPTL4 upregulation, mitigates weight loss, and reduces lung edema in a single-infection model, as assessed on day 7 post-infection.**

**A.** ANGPTL4 protein levels were markedly elevated following PR8 influenza infection and significantly reduced upon *Angptl4*-ASO treatment, demonstrating effective target knockdown in the viral setting (*n* = 5 per group). Data are shown as mean ± SD. Significance was analyzed by one-way ANOVA followed by Tukey’s test (***p* < 0.01, ****p* < 0.001).

**B.** Body weight trajectories in the PR8 infection model. PR8-infected mice displayed progressive weight loss, whereas PR8-ASO mice showed significantly attenuated weight loss over the 7-day period (*n* = 5 per group). Data are shown as mean ± SD. Significance was analyzed by one-way ANOVA followed by Tukey’s test (****p* < 0.001, *****p* < 0.0001).

**C.** Quantification of the W/D lung weight ratio. PR8 infection induced substantial lung edema, which was markedly alleviated by *Angptl4*-ASO administration. Data are shown as mean ± SD. Significance was analyzed by two-way ANOVA followed by Tukey’s test (**p* < 0.05).


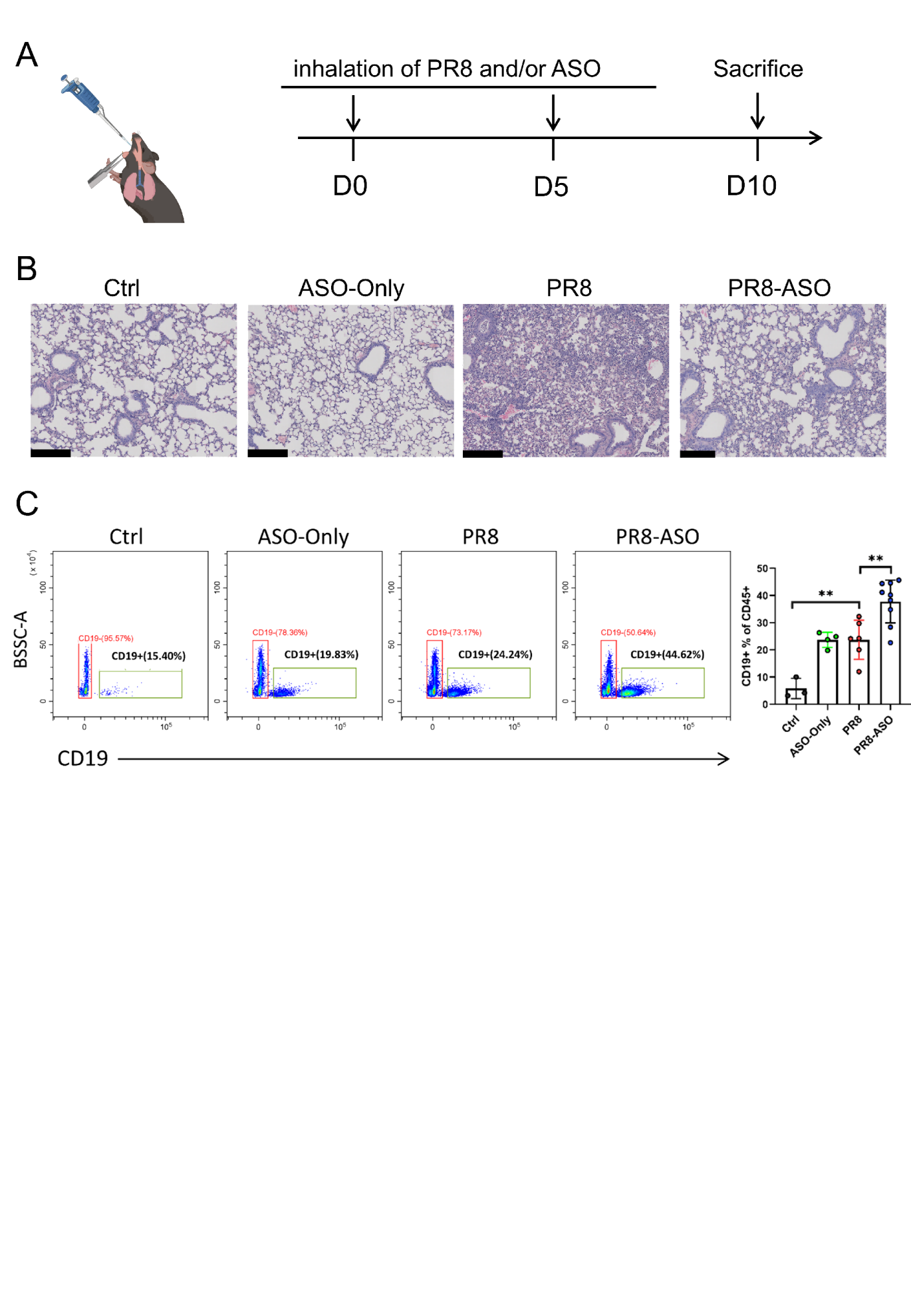


**Figure S5. Repeated inhaled delivery of *Angptl4*-ASO mitigates lung inflammation and alters immune cell composition in mice that received repeated PR8 infection.**

**A.** Experimental timeline of the PR8 influenza repeated infection model. Mice received inhaled PR8 and/or *Angptl4*-ASO on Day 0 and Day 5, followed by sacrifice on Day 10.

**B.** Representative H&E-stained lung sections. Ctrl and ASO-only groups exhibited normal alveolar architecture without inflammatory infiltration. PR8 infection caused marked alveolar destruction, interstitial thickening, and dense accumulation of inflammatory cells. PR8-ASO treatment substantially preserved lung structure and reduced inflammatory infiltration (scale bar: 100 μm).

**C**. Flow cytometric analysis of lung single-cell suspensions. PR8 infection increased the frequency of CD19⁺ B cells within the CD45⁺ compartment, and *Angptl4*-ASO treatment further enhanced CD19⁺ cell proportions, consistent with modulation of the adaptive immune response during the resolution phase of infection. Quantification of CD19⁺ cells as a percentage of CD45⁺ leukocytes is shown on the right.

Data are presented as mean ± SD (*n* = 3–9 per group). Statistical analysis was performed using one-way ANOVA followed by Tukey’s test (***p* < 0.01).


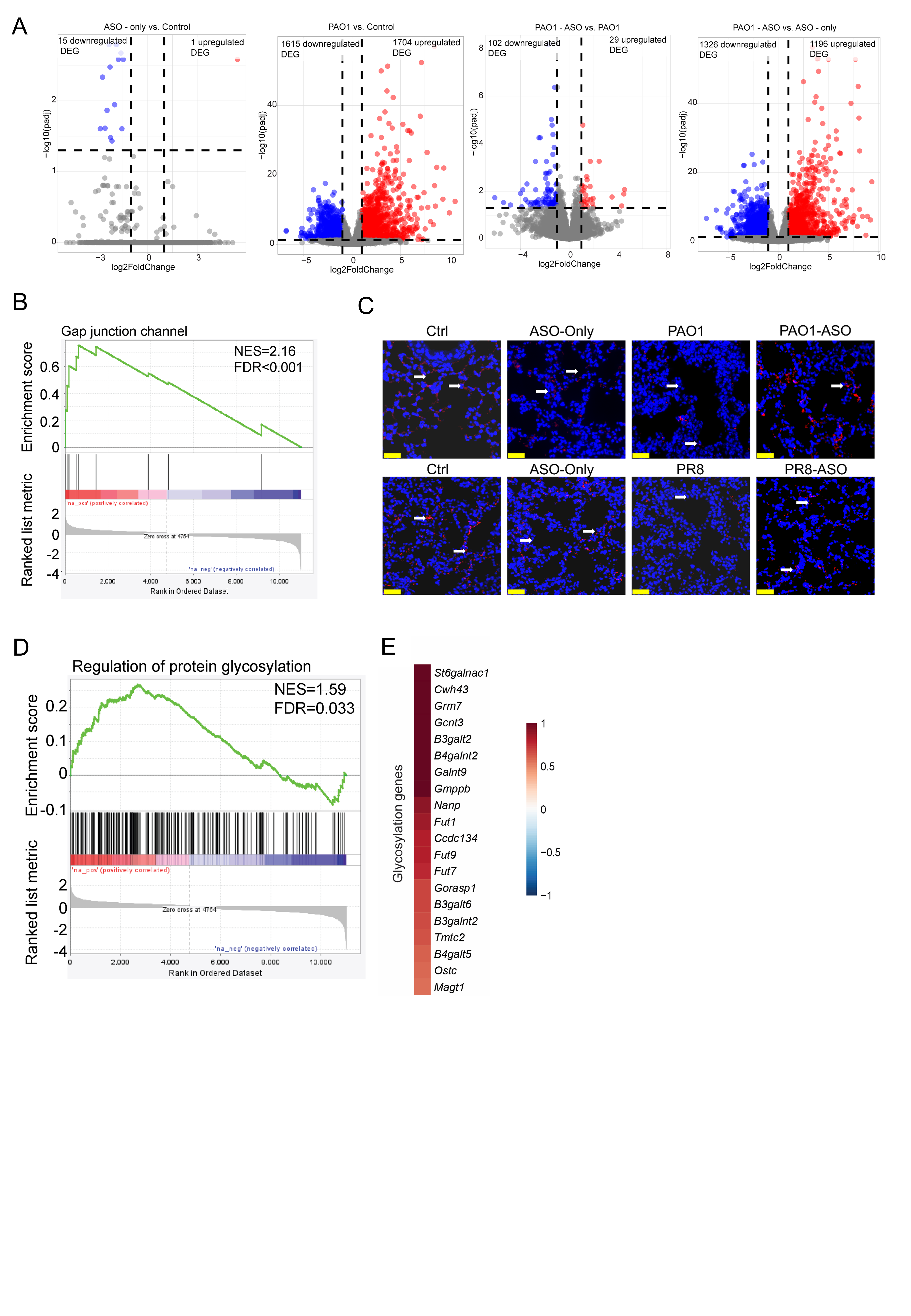


**Figure S6. *Angptl4*-ASO shows minimal off-target transcriptional effects and preserves tight junction integrity in single-infection models.**

**A.** Volcano plots comparing differentially expressed genes (DEGs) between treatment groups in the PAO1 acute infection model as analyzed on day 2 post-infection. Significantly upregulated genes are shown in red, downregulated genes in blue, and nonsignificant genes in grey. The x-axis indicates log₂ fold change, and the y-axis represents –log₁₀ (adjusted *p*-value). ASO-only treatment induced minimal transcriptional alterations relative to control, whereas PAO1 infection caused broad gene dysregulation. Co-treatment with *Angptl4*-ASO substantially reduced the magnitude of PAO1-induced transcriptional disturbances.

**B.** Enrichment plots of gene sets related to Gap junction channel of PAO1-ASO group at day 2 post-PAO1 infection.

**C.** Immunofluorescence staining of ZO-1 (red) in lung sections from PAO1 (day 2 post-infection) and PR8 (day 7 post-infection) infection models. Nuclei were counterstained with DAPI (blue). Uninfected control (Ctrl), ASO-only, and ASO-treated infected groups (PAO1-ASO, PR8-ASO) exhibited strong ZO-1 localization along bronchial epithelium, alveolar septa, and surrounding interstitial regions (white arrows). In contrast, PAO1- and PR8-infected (ASO untreated) mice displayed markedly diminished ZO-1 expression, restricted to limited epithelial areas (scale bar: 20 μm).

**D.** Enrichment plots of gene sets related to the regulation of protein glycosylation of PAO1-ASO group at day 2 post-PAO1 infection.

**E.** Heatmap of the differential expression of genes related to the regulation of protein glycosylation, showing PAO1-ASO / PAO1 group values at day 2 post-PAO1 infection. Red indicates higher expression in the PAO1-ASO group; blue indicates lower expression.


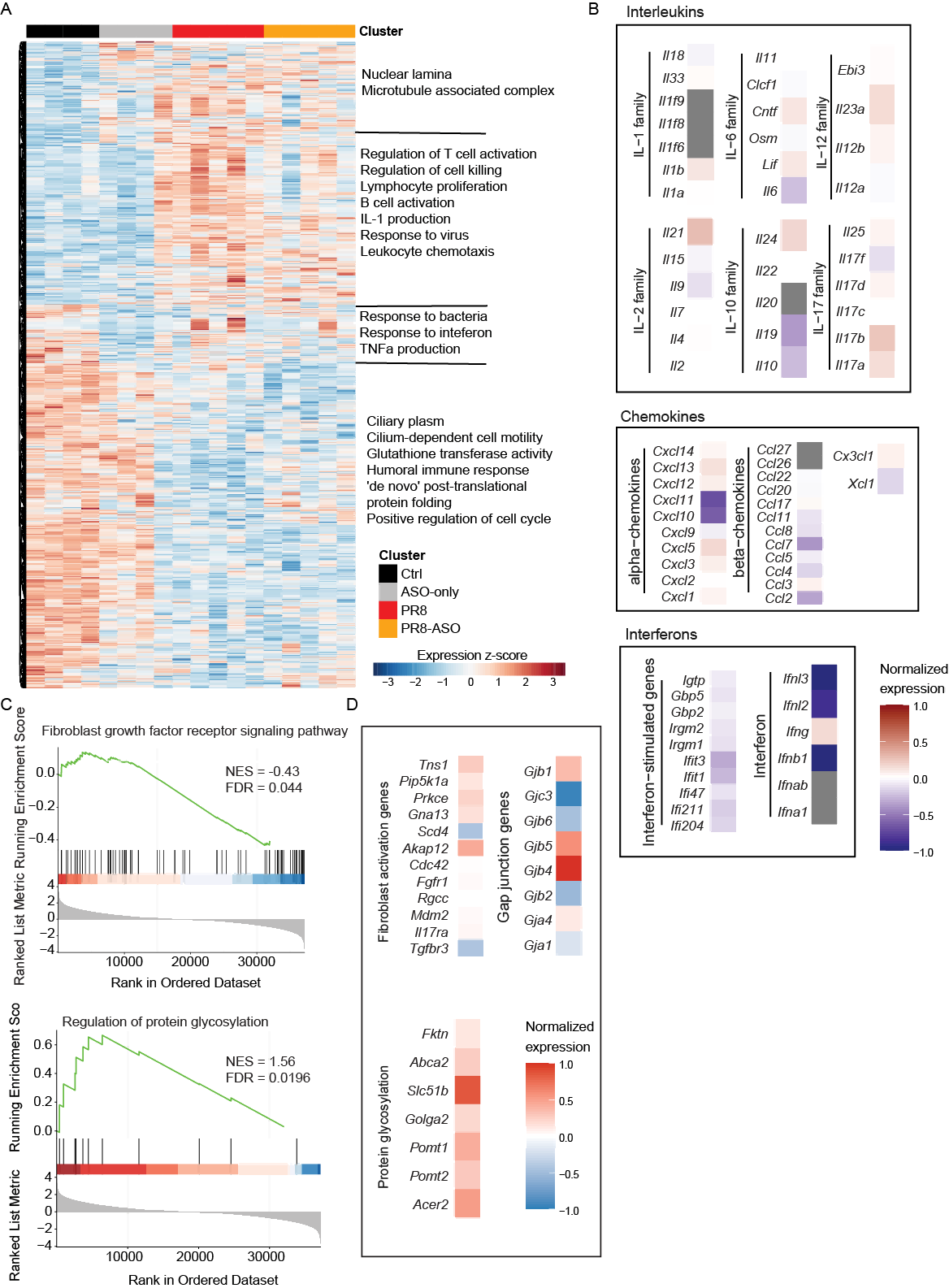


**Figure S7.** **Transcriptome analysis reveals molecular activities of *Angptl4*-ASO in a PR8 single-infection model, analyzed on day 7 post-infection.**

**A.** Heatmap illustrating DEGs in between treatment groups. Each row represents a gene, and each column a sample. Red indicates higher expression levels and blue indicates lower expression levels (*n* = 4 for Ctrl and ASO-only groups; *n* = 5 for PR8 and PR8-ASO groups).

.

**B.** Heatmap of the differential expression of interleukins, chemokines, and interferons between *Angptl4*-ASO treated and untreated mice infected with PR8. The proteins are grouped based on their molecular families. Red indicates higher expression while purple indicates lower expression in PR8-ASO relative to PR8 mice.

**C.** Enrichment plots of gene sets related to fibroblast growth factor (FGF) receptor signaling pathway and regulation of protein glycosylation of the PR8-ASO group.

**D.** Heatmap showing the differential expression of genes related to fibroblast activation, gap junction, and protein glycosylation in PR8-ASO versus PR8-infected (untreated) mice. Red indicates higher expression in the PR8-ASO group; blue indicates lower expression.

.


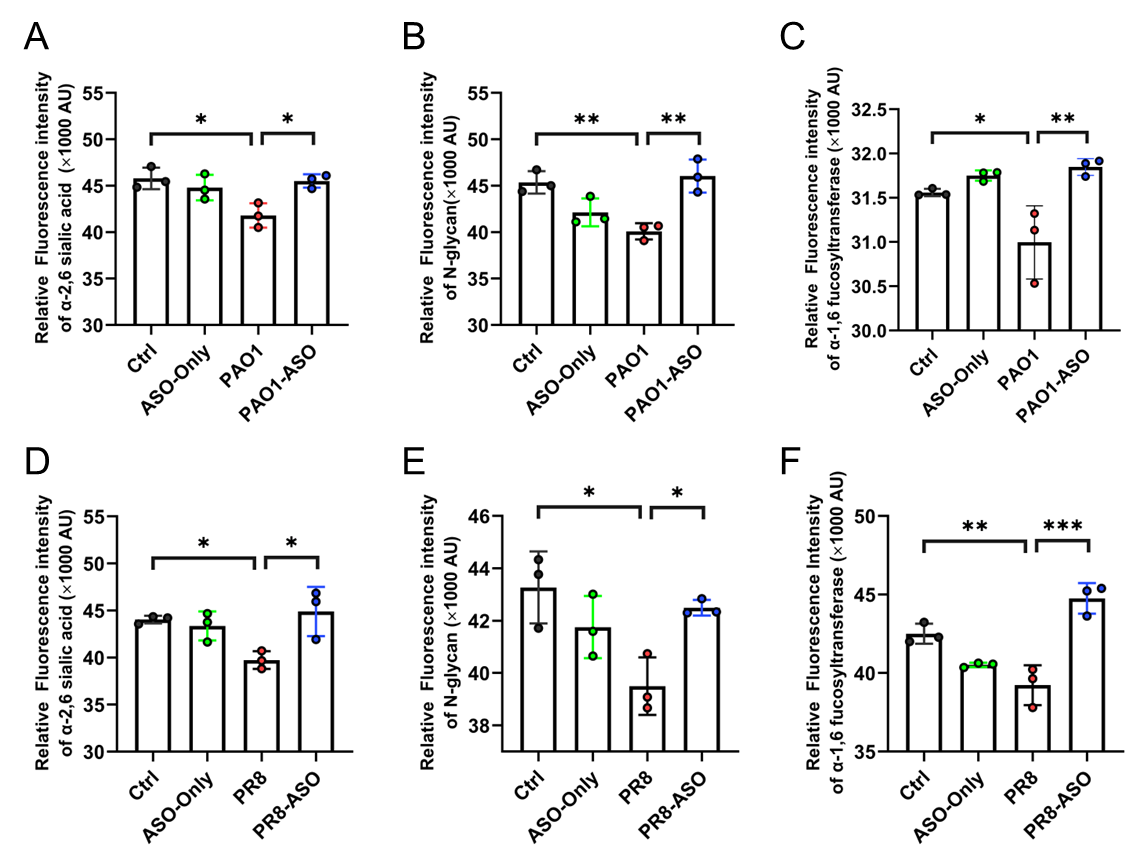


**Figure S8. *Angptl4*-ASO restores infection-induced loss of epithelial glycoproteins in PAO1 and PR8 single-infection lung injury models.**

**A-C.** Relative fluorescence intensity of key glycoproteins in bronchial and alveolar epithelium of the PAO1 acute infection model at day 2 post-infection. PAO1 infection significantly reduced the levels of A) α-2,6 sialic acid, B) N-glycan, and C) α-1,6 fucosyltransferase, and the *Angptl4*-ASO treatment reversed this reduction.

**D-F.** Relative fluorescence intensity of key glycoproteins in bronchial and alveolar epithelium of the PR8 infection model at day 7 post-infection. PR8 infection induced a similar decrease in D) α-2,6 sialic acid, E) N-glycan, and F) α-1,6 fucosyltransferase, which was likewise restored by ASO treatment.

Data are presented as mean ± SD (*n* = 3 per group). Statistical analysis was performed using one-way ANOVA followed by Tukey’s test (**p* < 0.05, ***p* < 0.01, ****p* < 0.001).

**
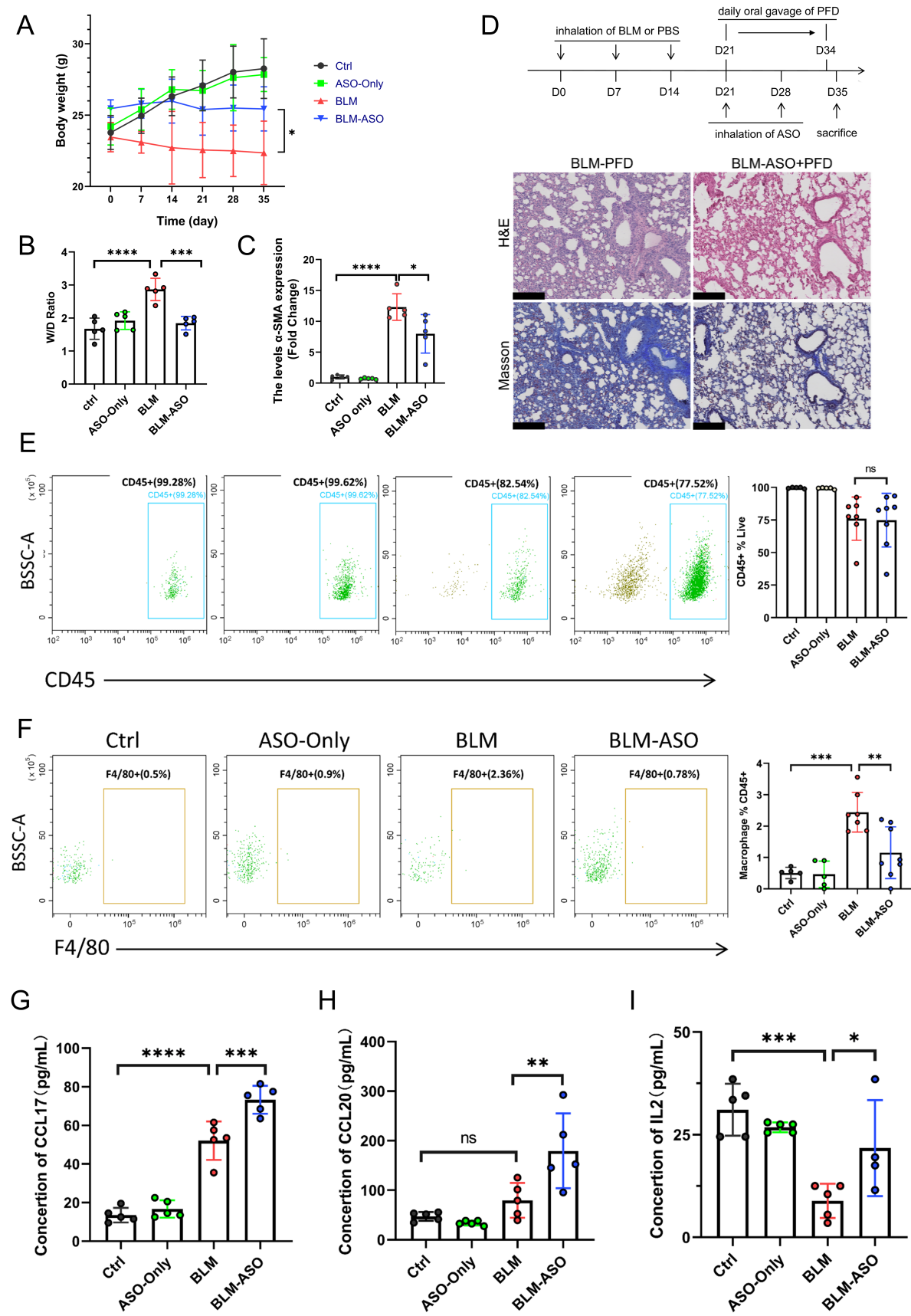
**

**Figure S9. *Angptl4*-ASO ameliorates bleomycin (BLM)-induced pulmonary fibrosis via immunomodulation at day 35 post-BLM treatment.**

**A.** Body weight trajectories in the BLM–induced pulmonary fibrosis model. *Angptl4*-ASO treatment improved body weight maintenance compared with BLM-treated mice (*n* = 5 per group). Data are shown as mean ± SD. Significance was analyzed by two-way ANOVA followed by Tukey’s test (**p* < 0.05).

**B.** Quantification of the W/D lung weight ratio. BLM induced substantial lung edema, which was markedly alleviated by *Angptl4*-ASO administration (*n* = 5 per group). Data are shown as mean ± SD. Significance was analyzed by one-way ANOVA followed by Tukey’s test (****p* < 0.001, *****p* < 0.0001).

1. Quantitative analysis of α-SMA immunofluorescence. α-SMA expression was reduced after ASO treatment (*n* = 5 per group). Data are shown as mean ± SD. Significance was analyzed by one-way ANOVA followed by Tukey’s test (**p* < 0.05, *****p* < 0.0001).

**D.** Representative images of H&E and Masson’s trichrome-stained lung sections from BLM-treated mice receiving pirfenidone (BLM-PFD) or combined *Angptl4*-ASO and pirfenidone therapy (BLM-ASO + PFD). Mice underwent inhaled administration of BLM or PBS once weekly for three consecutive weeks. From Day 21 to Day 34, mice in the monotherapy group received daily PFD via oral gavage, while those in the combination group received both daily oral PFD and inhaled ASO on Day 21 and Day 28. The combination treatment markedly attenuated alveolar wall thickening, inflammatory infiltration, and collagen deposition compared with PFD monotherapy, indicating enhanced antifibrotic efficacy (scale bar: 200 μm).

**E-F.** Flow cytometric analysis of BALF. The overall percentage of CD45⁺ leukocytes among live cells remained comparable across groups (E). In contrast, BLM treatment significantly increased the proportion of F4/80⁺ macrophages within the CD45⁺ compartment, whereas *Angptl4*-ASO reduced macrophage abundance toward baseline levels (F). (*n* = 5 for Ctrl and ASO-only groups; *n* = 7 for BLM group; and *n* = 8 for BLM-ASO group). Data are shown as mean ± SD. Significance in was analyzed by Kruskal-Wallis test with Dunn’s post-hoc test (E), or one-way ANOVA followed by Tukey’s test (***p* < 0.01, ****p* < 0.001, ns, *p* > 0.05)

1. **I**. Concentrations of anti-inflammatory cytokines G) CCL17, H) CCL20, and I) IL-2 in BALF. Levels of these mediators were significantly higher in BLM-ASO mice than in the BLM group, indicating that *Angptl4*-ASO promotes an anti-inflammatory milieu in the fibrotic lung (*n* = 4 or 5 per group). Data are shown as mean ± SD. Significance was analyzed by one-way ANOVA followed by Tukey’s test (**p* < 0.05, ***p* < 0.01, ****p* < 0.001, *****p* < 0.0001, ns, *p* > 0.05).


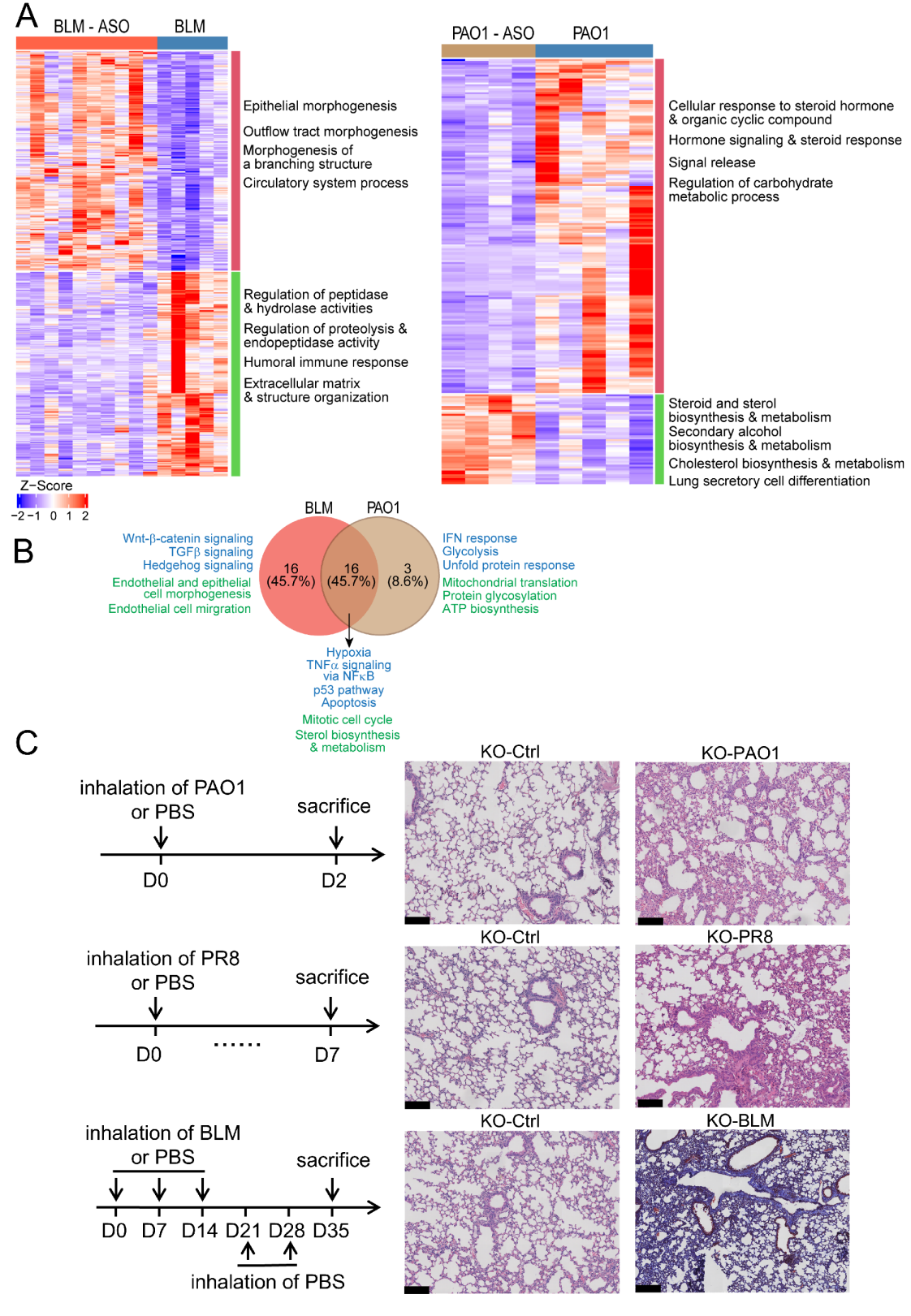


**Figure S10. Comparative transcriptomic analysis of *Angptl4*-ASO treatment in the day 2 post-PAO1 infection model and the day 35 post-BLM treatment model.**

**A.** Heatmap illustrating significantly altered biological processes in PAO-induced acute lung injury and BLM-induced pulmonary fibrosis following *Angptl4*-ASO treatment. Data represent normalized *z*-scores, highlighting distinct and shared biological responses (*n* = 10 for the BLM-ASO group; n = 5 for the BLM group; n = 4 for the PAO1-ASO group; n = 5 for the PAO1 group).

**B.** Venn diagram illustrating the overlap and distinct biological hallmarks significantly altered by *Angptl4*-ASO treatment in PAO1-induced acute lung injury and BLM-induced pulmonary fibrosis models. Approximately 45.7% (16 hallmarks) were shared between both models, including hypoxia, TNFα-NFκB signaling, apoptosis, p53-mediated responses, mitotic cell-cycle regulation, and sterol biosynthesis/metabolism, indicating conserved therapeutic mechanisms. Model-specific pathways, such as Wnt-β-catenin/TGFβ signaling and endothelial-epithelial morphogenesis in the BLM model, and IFN response, mitochondrial translation, and ATP biosynthesis in the PAO1 model, reflect the context-dependent modulation of ANGPTL4 activity across distinct lung injury contexts.

**C.** Lung pathology in *Angptl4-*knockout (KO) mice following PAO1, PR8, or BLM challenge. In the PAO1 model, KO mice were administered with PAO1 or PBS on Day 0 and euthanized on Day 2. In the PR8 model, KO mice were administered with PR8 or PBS on Day 0 and euthanized on Day 7. In the BLM model, KO mice were administered with BLM or PBS on Days 0, 7, and 14, followed by PBS on Days 21 and 28; all mice were euthanized on Day 35. H&E staining revealed markedly attenuated inflammatory injury in infected KO mice, while Masson's trichrome staining showed reduced collagen deposition and fibrosis in BLM model, compared to their wildtype counterparts (scale bar: 100 µm).


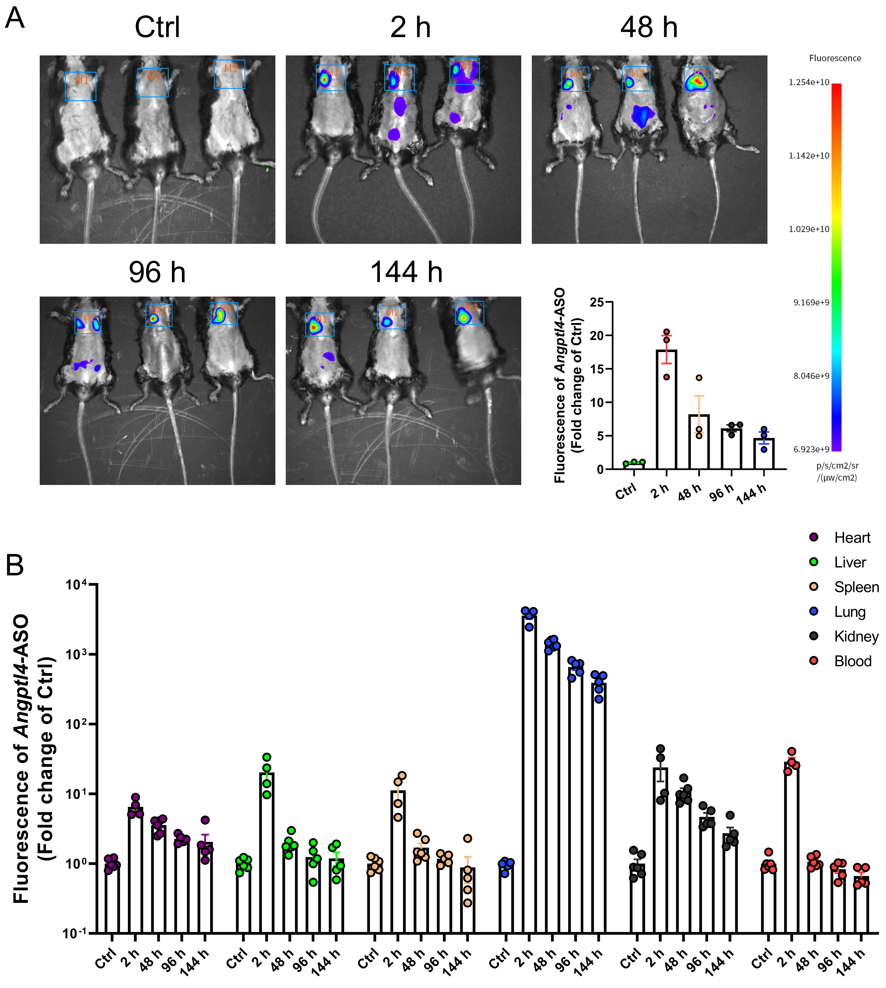


**Figure S11. Biodistribution and retention of Cy5-labeled *Angptl4*-ASO in the mouse lung following inhalation administration.**

**A.** *In vivo* fluorescence images of mice at various time points (2, 48, 96, and 144 hours) after inhalation of Cy5-*Angptl4*-ASO. The control group mouse, which did not receive the ASO, is shown for comparison. Strong and persistent fluorescent signals are localized specifically to the thoracic region (lung), indicating effective delivery and prolonged retention of the ASO in the target tissue.

**B.** Quantitative analysis of Cy5 fluorescence intensity in major organs and blood at the indicated time points. Fluorescence intensity is expressed as the fold change relative to the control group. The data confirm the predominant accumulation and extended presence of the ASO in the lungs, with minimal distribution to other off-target organs.
